# Dasatinib reverses Cancer-associated Fibroblasts (CAFs) from primary Lung Carcinomas to a Phenotype comparable to that of normal Fibroblasts

**DOI:** 10.1186/1476-4598-9-168

**Published:** 2010-06-27

**Authors:** Silke Haubeiss, Jens O Schmid, Thomas E Mürdter, Maike Sonnenberg, Godehard Friedel, Heiko van der Kuip, Walter E Aulitzky

**Affiliations:** 1 Dr. Margarete Fischer-Bosch Institute of Clinical Pharmacology and University of Tuebingen, Auerbachstr. 112, 70376 Stuttgart, Germany; 2 Klinik Schillerhöhe, Department of Thoracic Surgery, Gerlingen, Germany; 3 2ndDepartment of Internal Medicine, Oncology and Hematology, Robert Bosch Hospital, Auerbachstr 110, 70376 Stuttgart, Germany

## Abstract

Cancer associated fibroblasts (CAFs) play a critical role for growth, invasion, and metastasis of cancer. Therefore, targeting CAFs with small molecule inhibitors may be an attractive anti-tumor strategy. The current study aims to identify small molecule kinase inhibitors affecting CAF's growth and to characterize the biological effects of active compounds on primary CAFs from lung cancer. We screened two individual CAF strains for their sensitivity to a panel of 160 kinase inhibitors. Five kinase inhibitors were identified inhibiting more than 50% of the growth of both cell lines. Three of them were inhibitors of PDGFR at nanomolar concentrations. Therefore, we further tested the FDA approved PDGFR inhibitors Dasatinib, Nilotinib, Sorafenib, and Imatinib. All 37 CAF strains investigated were highly sensitive to Dasatinib at clinically relevant concentrations. Imatinib was slightly less effective, whereas the inhibitory effects of Nilotinib and Sorafenib were significantly less pronounced.

We investigated the effect of Dasatinib on the CAF transcriptome by microarray analysis of 9 individual CAF strains. 492 genes were identified whose expression was changed at least twofold. 104 of these encoded cell cycle related proteins with 97 of them being downregulated by Dasatinib. The majority of regulated genes, however, were of diverse biological functions not directly related to proliferation. We compared this Dasatinib expression signature to previously described differential signatures of normal tissue associated fibroblasts (NAFs) and CAFs and to a signature of fibroblast serum response. There was a significant overlap between genes regulated by Dasatinib and serum repression genes. More importantly, of the 313 genes downregulated by Dasatinib 64 were also reduced in NAFs compared to CAFs. Furthermore, 26 of 179 genes identified as upregulated by Dasatinib were also found to be elevated in NAFs compared to CAFs. These data demonstrate that Dasatinib partially reverses the phenotype of CAFs to a normal fibroblast like phenotype. This is further supported by the finding that incubation of tumor cells with conditioned medium from CAFs pre-incubated with Dasatinib significantly reduced tumor cell proliferation, suggesting that Dasatinib partially reverses the CAF mediated tumor promoting effect. Therefore, targeting CAFs with Dasatinib represents a promising therapeutic principle.

## Findings

The tumor microenvironment or "stroma" actively participates in tumorigenesis, tumor progression, and metastasis. Within the tumor stroma, CAFs are of outstanding importance. CAFs are the primary cell type that produces ECM and thereby determines dynamics of the tumor [1].

CAFs show a molecular and functional phenotype that is different from NAFs. It has been early recognized by pathologists that in many tumors stroma is characterized by an increased fibroblast proliferation [2]. CAFs also secrete a variety of growth factors and proteinases facilitating tumor growth and invasion [3-5]. The protective and supportive effects of CAFs on tumor cells strongly support the concept that CAFs represent an attractive target for anticancer therapy. The activity of a plethora of kinases is involved in signalling pathways important for the tumor promoting activities of CAFs including receptors for PDGF and TGFβ and corresponding downstream signal transducers [1]. Therefore, we investigated the potency of kinase inhibitors to block CAF activities.

**Figure 1:**
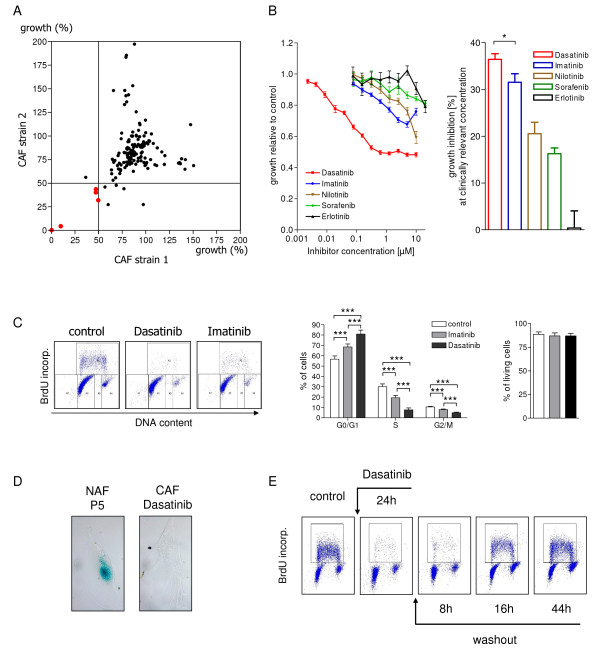
**PDGFR inhibitors block proliferation of CAFs without inducing cell death**. ***A***, CAFs from two lung adenocarcinomas were cultivated as described [27] and incubated with 160 different kinase inhibitors (1 μM each). After 48 hours the efficacy of the inhibitors was monitored by MTT. Points represent mean values from two experiments (relative to DMSO-treated controls). Red points represent growth reduction >50% in both strains. ***B***, Left panel: MTT experiments performed with indicated inhibitor concentrations. Data represent mean ± SEM from 37 (Dasatinib, Imatinib), 21 (Nilotinib), 7 (Sorafenib), and 10 CAF strains (Erlotinib). Experiments were performed in triplicates. Right panel: MTT experiments with one concentration representing Cmax. Statistics was performed using unpaired students t-test (*: p < 0.05). ***C***, Proliferation analyzed by BrdU labelling and PI staining. Left panel: representative experiment. Middle panel: percentages of cells in G0/G1, S, and G2/M from CAFs incubated with/without Imatinib (3 μM) or Dasatinib (0.1 μM) for 24 hours (mean ± SEM from 11 strains. Statistics was performed with paired student's t-test; ***: p < 0.001). Right panel: Annexin V staining of CAFs following Imatinib or Dasatinib for 24 hours (mean ± SEM from 11 strains). ***D***, CAFs were stained for β-galactosidase 7 days after treatment with 0.1 μM Dasatinib for 48 hours. As a control we used NAFs cultivated for 5 passages (representative examples). ***E***, Proliferation of CAFs following Dasatinib washout. Cells were treated with Dasatinib for 24 hours. Dasatinib was then washed out and cells were cultivated in drug-free medium for indicated times. Samples were analyzed by BrdU labelling and PI staining (representative experiment).

We screened a library of 160 kinase inhibitors at low concentration (Additional file 1, Material and Methods) for their effect on proliferation and viability of CAFs isolated from 2 primary lung cancer specimens (Figure 1A). The most potent compounds with a growth inhibition of more than 50% in both tested CAF strains turned out to be Staurosporine (broad spectrum inhibitor also inhibiting PDGFR), one PDK1/Akt/Flt inhibitor, K-252a (inhibiting CaM kinase, PKC/PKA, and PDGFR at 100 nM), PI-103 (PI3K/mTOR inhibitor), and one specific PDGFR inhibitor (Additional file 2, Table S1). Thus, 3 of these 5 active inhibitors antagonize PDGFR activity at nanomolar concentrations. These data show that, even under conditions of high serum concentrations, PDGFR signaling is a critical pro-proliferative stimulus for CAFs *in vitro*. This finding is in accordance with earlier studies demonstrating that PDGF plays a central role in desmoplasmic reaction in a breast cancer xenograft model [6]. While most epithelial tumor cells lack the PDGFR but secrete PDGF, PDGFR is frequently found on CAFs and expression of activated PDGFR is associated with metastatic potential in colon carcinomas [7]. Targeting PDGFR may therefore be a powerful strategy to inhibit the activated phenotype of CAFs and consequently reduce their ability to promote and support tumor cells. This is supported by a limited number of studies showing that PDGFR inhibition by Imatinib not only reduces fibroblast proliferation *in vitro *[8] but also slows progression of cervical carcinomas [9], growth of pancreatic carcinomas [10], progression and metastasis of colon carcinomas [7], and improves the uptake of radioimmunotherapy in colorectal carcinomas [11]*in vivo*. More recently, Nilotinib and Dasatinib, two additional inhibitors of Abl and PDGFR kinases have also been approved for treatment of CML [12,13]. In analogy to Imatinib, both inhibitors have been shown to affect fibroblast growth [14].

As a next step, we studied the effects of four FDA approved inhibitors Dasatinib, Imatinib, Nilotinib, and Sorafenib, which all target PDGFR *in vitro*, in a panel of CAF strains from individual lung cancer patients. All four inhibitors blocked CAF growth whereas the EGFR inhibitor Erlotinib, which was used in control experiments, was inactive at clinically relevant concentrations (Figure 1B). However, despite the fact that Dasatinib, Imatinib, Nilotinib, and Sorafenib inhibit PDGFR with comparable IC50 values ranging from 28 nM - 80 nM [15-17], their effects on proliferation and/or viability of CAF strains from lung carcinomas *in vitro *were remarkably different. Dasatinib was highly efficient in reducing CAF growth already at concentrations below 100 nM (Figure 1B, left panel) whereas approximately 10fold higher inhibitor concentrations were required to achieve a comparable growth reduction by Imatinib, Nilotinib, and Sorafenib (Figure 1B, left panel). The most likely explanation for the diverging biological activities of these PDGFR-inhibitors is the different spectrum of kinases targeted in addition to PDGFR. In contrast to Imatinib, Nilotinib, and Sorafenib, Dasatinib targets a variety of other kinases such as Src kinases, TEC kinases, MAP kinases and others [18]. Combined inhibition of PDGFR together with blocking of intracellular signalling cascades may be more effective than inhibition of PDGFR alone.

Highly different plasma concentrations are achieved in pharmacokinetic studies with Imatinib, Dasatinib, Nilotinib, and Sorafenib [19-22]. To estimate whether the differing *in vitro *activity of these compounds can also be expected after *in vivo *exposure we compared inhibitor concentrations corresponding to Cmax values observed in clinical studies. All CAFs responded to Dasatinib at a concentration of 0.11 μM with a reduction of cell growth by 36.4 ± 1.2% (mean ± SEM). Imatinib was slightly less effective (31.5 ± 1.8% at 5.3 μM), whereas the inhibitory effects of Nilotinib and Sorafenib were significantly less pronounced with a reduction of cell growth by 20.5 ± 2.4% and 16.3 ± 1.2%, respectively (Figure 1B). We then studied the mechanism of action of the most effective compounds Dasatinib and Imatinib. Both molecules primarily reduced the fraction of S phase cells as indicated by a significant reduction of DNA synthesis. The effect of Dasatinib on inhibition of proliferation was more pronounced than that of Imatinib (74.5 ± 5.9% versus 35.8 ± 7.5%; Figure 1C). No induction of cell death was seen under these conditions (Figure 1C). Moreover, Dasatinib did not induce senescence in CAFs as β-galactosidase staining was not evident in Dasatinib treated CAFs (Figure 1D). This is further supported by the finding that the Dasatinib-induced proliferation stop is reversible since cells were able to re-enter the cell cycle, synthesize DNA, and proliferate after removal of Dasatinib (Figure 1E). Therefore, Dasatinib represents a highly active compound to block proliferation in CAFs.

**Figure 2:**
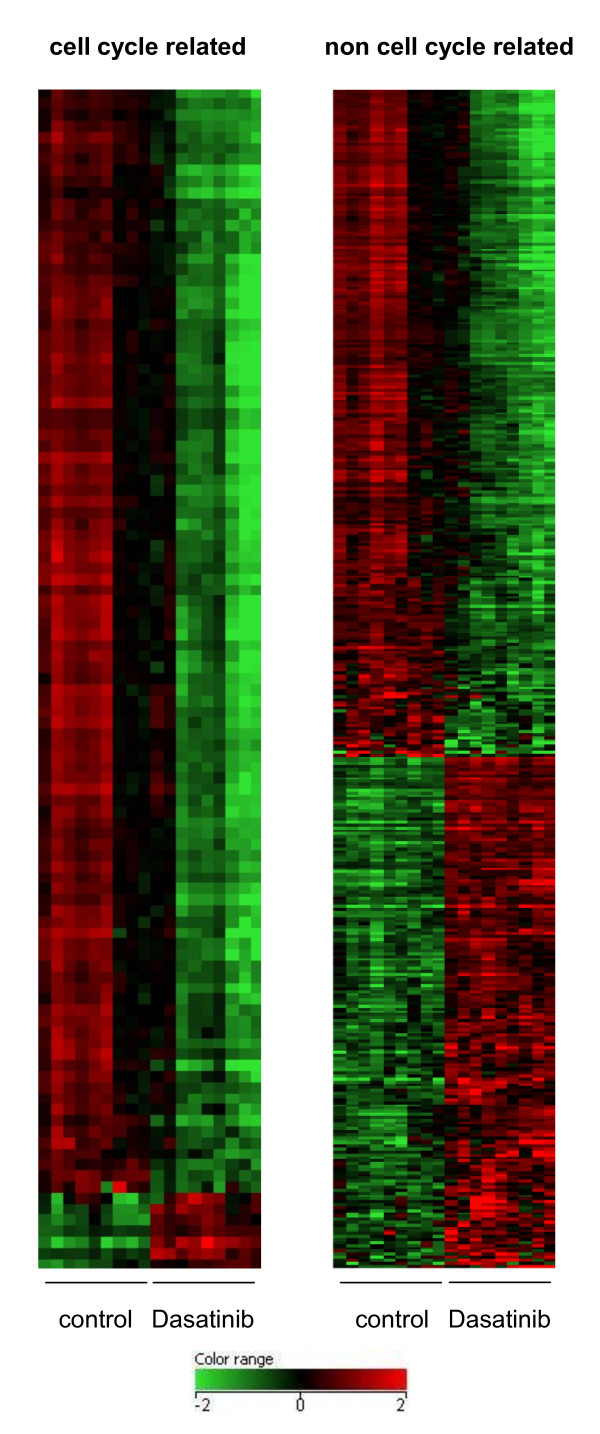
**Effect of Dasatinib on gene expression**. Heatmap of transcripts regulated by Dasatinib. Cell cycle related transcripts are shown in the left panel and transcripts not related to cell cycle in the right panel. Nine CAF strains isolated from 9 different lung carcinoma specimens were incubated with or without Dasatinib for 48 hours and analyzed with microarrays. Transcripts regulated by Dasatinib were identified by a significant 2fold change. The relative expression levels for a gene among the samples are indicated by green for low value and by red for high value. Each row represents a transcript; each column represents one sample (see also: Additional file 3, Table S2).

As Dasatinib appeared to be the most effective compound we characterized its molecular effects by performing microarray analysis (Additional file 1, Material and Methods) of nine individual CAF strains cultivated in presence or absence of Dasatinib. We identified 511 transcripts (492 genes) whose expression was changed significantly at least twofold (Figure 2A; Additional file 3, Table S2). Gene ontology analysis identified 107 of these (104 genes) as cell cycle related of whom 97 were downregulated (e.g. *CDK2*, *FOXM1*, *CDC20*, *E2F7*, *MKI67*). Of the remaining 404 transcripts (388 genes), 229 transcripts (216 genes) were downregulated in the presence of Dasatinib. Among these we found additional genes coding for proteins important for the control of DNA integrity such as DNA repair proteins (e.g. *FANC *genes, *BRCA1/2*, *RAD51*, *XRCC2*). 175 transcripts (172 genes) were upregulated such as genes encoding for receptors (*PDGFRB*, *ROR1*, *CRYAB*), ECM and adhesion proteins (such as *MCAM*, Integrins, Laminins, Collagens).

**Figure 3:**
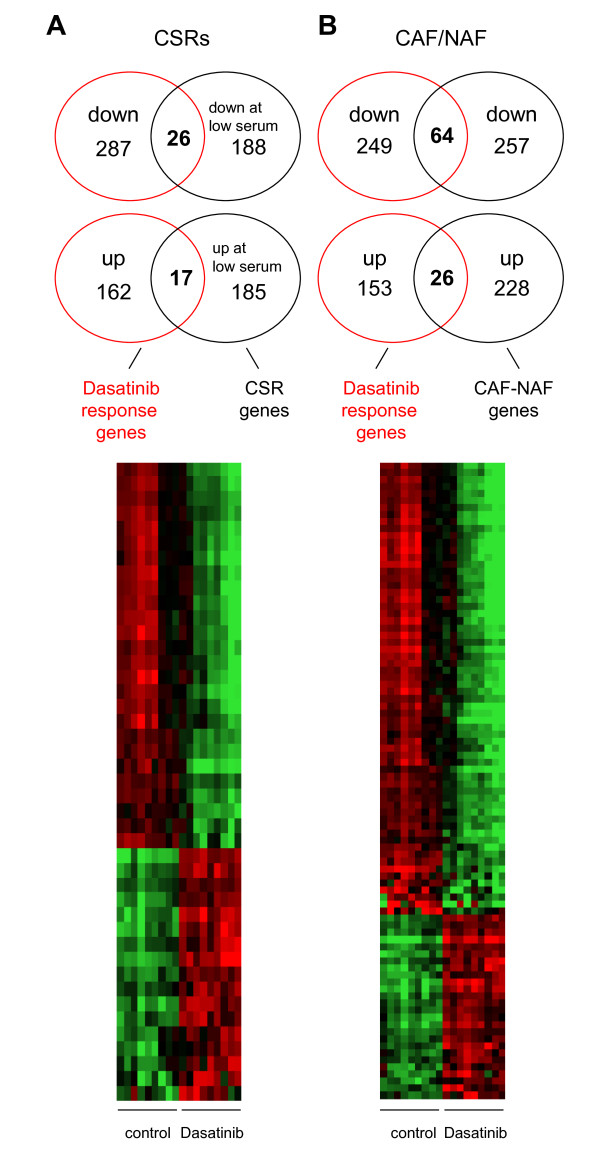
**Relationship of Dasatinib regulated genes with functional datasets resembling quiescence **[24]**and normal fibroblasts **[26]. ***A***, Venn diagrams depicting the number of genes differentially regulated by Dasatinib only, by Dasatinib and serum, and by serum only (upper panel). Both the overlap of genes downregulated and upregulated in both datasets is highly significant (p = 1.5 × 10^-17 ^and p = 2.0 × 10^-12^; Fisher's exact test). The lower panel represents the heatmap of the overlap between Dasatinib regulated genes and core serum response genes. ***B***, Venn diagrams depicting the number of genes regulated by Dasatinib only, differentially expressed in CAFs in absence or presence of Dasatinib and in CAFs vs. NAFs, and genes differentially expressed in CAFs vs. NAFs only (upper panel). The overlap of genes downregulated and upregulated in both datasets is highly significant (p = 6.6 × 10^-30 ^and p = 2.3 × 10^-11^; Fisher's exact test). Heatmap of expression patterns of Dasatinib regulated genes overlapping with genes differentially regulated in CAFs vs. NAFs (lower panel).

Several transcriptome signatures related to the function of NAFs and CAFs have been reported. As expected, a significant overlap between the Dasatinib response genes and genes associated with quiescence in normal fibroblasts exists [23] (Additional file 4, Table S3). More importantly, a quiescence-associated expression pattern could recently be identified to be of prognostic value in epithelial tumors [24,25]. These authors defined core serum response genes not related to cell cycle processes in fibroblasts as genes differentially regulated upon addition of serum to the culture medium. Dasatinib downregulated 26 of the 214 serum induced genes. Accordingly, Dasatinib upregulated 17 of the 202 genes whose expression was blocked by addition of serum (p < 10^-12^; Fisher's exact test). Thus, a significant number of genes were regulated by Dasatinib treatment in the same direction as by growth factor withdrawal (Figure 3A; Additional file 5, Table S4). This is in line with the observation that Dasatinib induces a quiescent state in CAFs.

However, many genes of the Dasatinib response signature are not overlapping with the serum-repressed pattern. This observation supports the view that Dasatinib induces more than a quiescence-like phenotype in fibroblasts. We therefore compared the Dasatinib response signature to the published differential expression pattern of NAFs and CAFs from breast cancer cases [26]. 64 of the genes found to be downregulated by Dasatinib were also lower expressed in NAFs compared to CAFs, while only 8 genes should be expected by chance alone (p < 10^-30^; Fisher's exact test). Furthermore, 26 genes were upregulated in CAFs treated with Dasatinib and also in NAFs (4 genes by chance alone; p < 10^-11^) (Figure 3B; Additional file 6, Table S5). We selected 6 genes from the 90 genes in the overlap for validation by qRT-PCR (Additional file 1, Material and Methods). Two of them were overexpressed (*PDGFR *and *SVEP1*). The expression of both could be confirmed to be significantly higher in Dasatinib treated CAFs by qRT-PCR. Four genes downregulated by Dasatinib and lower expressed in NAFs (*MMP1*, *MKI67*, *TTK*, and *FOXM1*) were confirmed to be reduced by qRT-PCR, reaching significance for *MMP1*, *TTK *and *FOXM1 *(Additional file 7, Figure S1).

**Figure 4:**
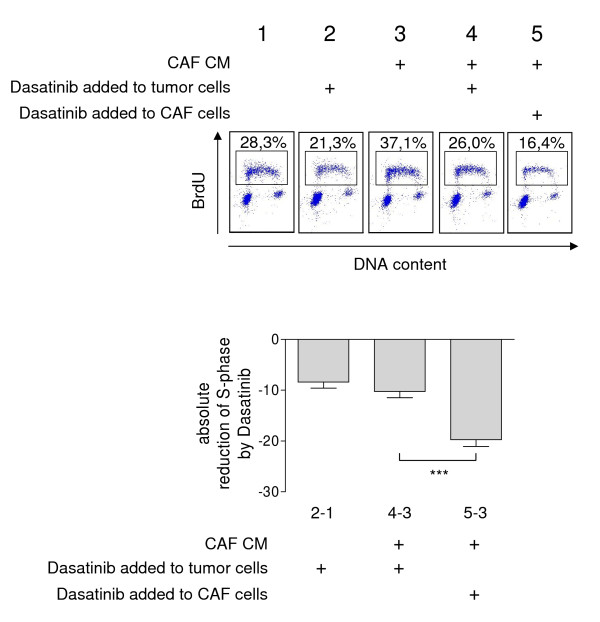
**Conditioned medium from Dasatinib-treated CAFs inhibits tumor cell proliferation**. H1299 epithelial tumor cells were cultivated with conditioned medium (CM; plots 3, 4, and 5) or control medium (plots 1 and 2) for 48 hours before harvesting for BrdU labelling and propidium iodide staining. CM was collected from CAFs cultivated in the presence or absence of Dasatinib for 48 h in medium supplemented with 0.1% FCS. Control medium (0.1% FCS) with or without Dasatinib was collected following incubation for 48 h without cells. Upper panel: representative result; lower panel: absolute reduction of the percentage of BrdU positive tumor cells in S phase upon Dasatinib. Dasatinib was either added to control medium in the absence of cells (first bar; 2 minus 1), to CAF conditioned medium after collecting CM (second bar; 4 minus 3), or to CAFs for 48 hours before collecting CM (third bar; 5 minus 3). Values reflect means ± SEM from 3 independent experiments (statistics was performed with paired student's t-test, 2-tailed, Holm corrected; ***: p < 0.001).

To assess whether the observed Dasatinib-mediated alteration of CAFs may influence tumor cell growth, we tested the effect of Dasatinib alone and that of conditioned medium (CM) from CAFs pre-incubated with or without Dasatinib on cell cycle and DNA synthesis of H1299 lung cancer cells. At low serum concentration CAF conditioned medium significantly enhanced tumor cell proliferation. This tumor promoting activity was completely abolished in CM from CAFs pre-treated with Dasatinib. Importantly, the reduction of tumor cell proliferation by CM from CAFs pre-treated with Dasatinib was significantly more pronounced than that observed with Dasatinib alone (Figure 4).

In conclusion, our data demonstrate that Dasatinib treatment partially reverses the CAF phenotype in fibroblasts from lung cancer tissues. More importantly, treatment of CAFs with Dasatinib reduces their ability to promote tumor proliferation *in vitro*. Treatment of lung cancer with Dasatinib may therefore be a promising strategy to enhance the efficacy of conventional therapy.

## List of abbreviations

μM: micro molar; BRCA1/2: breast cancer susceptibility gene 1/2; CAF: cancer associated fibroblast; CDC20: cell division cycle 20; CDK2: cycline dependent kinase 2; CM: conditioned medium; CRYAB: crystallin, alpha B; E2F7: E2F transcriptionfactor 7; ECM: extracellular matrix; EGFR: epithelial growth factor receptor; FANC: Fanconi; FOXM1: forkhead box M1; MCAM: melanoma cell adhesion molecule; MKI67: antigen identified by monoclonal antibody Ki-67; MMP1: matrix metallopeptidase 1; mTOR: mammalian target of rapamycin; NAF: normal tissue associated fibroblast; PDGF: platelet derived growth factor; PDGFR: platelet derived growth factor receptor; PDK1: pyruvate dehydrogenase kinase 1; PI: propidium iodide; PI3K: phosphoinositide-3 kinase; PKC/PKA: protein kinase C/protein kinase A; qRT-PCR: quantitative RT-PCR; ROR1: receptor tyrosine kinase-like orphan receptor 1; SVEP1: sushi, von Willebrand factor type A, EGF and pentraxin domain containing 1; TTK: TTK protein kinase; XRCC2: X-ray repair complementing 2.

## Competing interests

The authors declare that they have no competing interests.

## Authors' contributions

SH, JS, and MS carried out the functional and molecular studies. SH, JS, MS, and HK isolated and cultivated primary fibroblasts from lung tissue samples. TEM, HK, GF, and WEA conceived and designed the study. SH, HK, and WEA drafted the manuscript. SH, JS, TEM, HK, and WEA performed the statistical analysis. All authors read and approved the final manuscript.

## Supplementary Material

Additional file 1 **Material and Methods**. Description of materials and methods used in the study Click here for file

Additional file 2 Table S1. Potent CAF inhibitors Click here for file

Additional file 3 Table S2. 492 genes regulated by Dasatinib Click here for file

Additional file 4 **Table S3. Overlap of genes regulated by Dasatinib and upon serum withdrawal **[23] Click here for file

Additional file 5 **Table S4. Overlap of genes regulated by Dasatinib with core serum response genes **[24] Click here for file

Additional file 6 **Table S5. Overlap of genes regulated by Dasatinib with genes differentially expressed in NAFs vs. CAFs **[26] Click here for file

Additional file 7 **Figure S1. qRT-PCR validation of 6 genes from the overlap of genes regulated by Dasatinib with differentially regulated genes in NAFs vs. CAFs **[26]. Upper panel: graphical presentation of expression array data for six differentially expressed genes selected for qRT-PCR validation. Mean fold change of nine CAF cultures upon Dasatinib treatment. Lower panel: Expression levels (delta C_T _values) of selected genes as assessed by qRT-PCR in eight CAF cultures. Significant differences in expression in Dasatinib treated CAFs and controls were found for PDGFR (p = 0.039), SVEP1 (p < 0.0001), MMP1 (p = 0.048), TTK (p = 0.003), and FOXM1 (p = 0.002). Click here for file
