# Supplementary material for: Dasatinib reverses Cancer-associated Fibroblasts (CAFs) from primary Lung Carcinomas to a Phenotype comparable to that of normal Fibroblasts
Source: Mol Cancer. 2010 Jun 27;9:168. doi: 10.1186/1476-4598-9-168 (PMC2907332; doi:10.1186/1476-4598-9-168)
Supplement: Additional file 1 — Material and Methods. Description of materials and methods used in the study [file 1476-4598-9-168-S1.DOC]

**Materials and Methods**

**Patients and cell strains**

Primary lung tumors or lung metastases were obtained from newly diagnosed lung cancer patients from the Klinik Schillerhöhe immediately after resection. The investigation was approved by the local ethics committee (project number 396/2005V) and informed consent was obtained from the patients. Cancer-associated fibroblasts were isolated and cultured as described previously [27]. The lung carcinoma cell line H1299 was cultivated in RPMI supplemented with 10% FCS.

# Reagents

Imatinib mesylate (Gleevec), Dasatinib (Sprycel), Nilotinib (Tasigna), Sorafenib (Nexavar), and Erlotinib (Tarceva) were provided by Research Chemicals inc. (North York, Canada Novartis, Inc.). A stock solution (10 µM) was prepared for each by dissolving the compounds in DMSO. A collection of commercial inhibitors of mammalian Ser/Thr, and Tyr kinases was assembled from 2 Calbiochem libraries (Calbiochem, Merck, Darmstadt, Germany; Cat. No. 539744 and 539745) and used at a final concentration of 1 µM each.

**Cellular viability assay**

CAF cells were seeded in flat-bottomed 96-well culture plates (6,000-10,000 per well), 24 h later cells were treated with kinase inhibitors (1 μM for kinase library screen and increasing concentrations for Imatinib, Dasatinib, Nilotinib, Sorafenib, and Erlotinib) or vehicle control (DMSO) for additional 48 h in RPMI supplemented with 20% serum. At the end of the incubation period, survival of cells was determined by the MTT assay as described previously [27]. Briefly, MTT was added to each well and samples were incubated for 2 h before lysing in formazan dissolving solution. Colorimetric intensity was quantified using an ELISA reader at 590 nm. Values were obtained after subtraction of matched blanks (medium only). The OD values of DMSO controls were taken as 100% and values for drug treatment are expressed as % of control. Each measurement was performed in duplicates (kinase library) or triplicates (dosage titration for Imatinib, Dasatinib, Nilotinib, Sorafenib, and Erlotinib).

# Detection of apoptosis

The translocation of phosphatidylserine residues from the inner to the outer side of plasma membrane was assessed by FITC-conjugated Annexin-V staining (Pharmingen, San Diego, CA). Cells were stained 24 hours after addition of Dasatinib or Imatinib. Staining was performed according to manufacturers` instructions and analyzed by flow cytometry (BD FACSCalibur).

**Serum-reduced CAF conditioned medium**

CAFs were seeded in RPMI supplemented with 20% FCS on 75-cm^2^ flask and allowed to reach confluence. Medium was then replaced with serum-reduced RPMI 1640 (0.1% serum). The cells were incubated in the presence or absence of Dasatinib at 37°C for a further 48 hours. The medium was then collected, centrifuged, and stored at −20°C for later use. The control medium (RPMI supplemented with 0.1% FCS) was incubated for 48 hours in the presence or absence of Dasatinib.

H1299 cells were seeded into a 24-well plate with 20,000 cells/well in RPMI 1640 supplemented with 10% FBS and incubated at 37°C overnight. The medium was then replaced with either serum-reduced RPMI 1640 with or without Dasatinib, serum-reduced medium conditioned by CAF cells pre treated with/without Dasatinib at 100 nM, or CAF conditioned medium (CM) to which Dasatinib was added after collecting CM. After 48 hr, H1299 cells were pulse treated with BrdU and harvested for BrdU and propidium iodide staining.

**Measuring cell proliferation**

To label proliferating cells, 10 µM BrdU was applied to cultures for 45 min before harvesting and fixation in 70% cold ethanol. Cells were then treated with 2N HCl, 5% Triton X-100. Samples were neutralized with 0.1 M sodium borate and washed in PBS/1% BSA/0.5% Tween. Cells were stained with anti-BrdU antibody (BD Immunocytometry Systems, San Jose, USA) and a FITC conjugated anti-mouse antibody. After co-staining with propidium iodide cells were analyzed on a FACSCalibur flow cytometer and the percentage of BrdU positive cells was calculated.

**Detection of cellular senescence**

CAFs were cultivated in the presence of 0.1 µM Dasatinib for 48 hours and cultivated for further 7 days in the absence of Dasatinib. As a control we used normal fibroblasts isolated from lung tissue cultivated for 5 passages. Beta galactosidase activity at pH 6, a known characteristic of senescent cells, was detected using a senescence beta galactosidase staining kit (Cell Signaling Technology, Beverly, USA). The assay was performed according to the manufacturers´ instructions.

**Microarray Analysis**

Microarray Analysis was performed with 9 individual CAF strains. Briefly, fibroblasts at passages 3-5 were seeded in cell culture flasks and incubated with or without 0.1 µM Dasatinib for 48 h. At time of harvesting control cells reached a maximum of 80%-90% confluency. The efficacy of Dasatinib treatment in these CAFs was tested by BrdU staining or MTT analysis (data not shown). Following Dasatinib treatment, cells were trypsinized and the pellets were washed and then frozen in liquid nitrogen. RNA was extracted using the RNeasy Mini kit (Quiagen, Hilden, Germany). Microarray analysis was performed by the Microarray Facility Tübingen using humangene 1.0st Array (Affymetrix). Signal values were RMA normalized using Genespring GX 10.0.2 (Agilent Technologies, Santa Clara CA). Significant changes were determined by use of paired t-Test and fold change analysis was performed (FC > 2, p < 0.05, Benjamini-Hochberg FDR correction). Gene Ontology Annotations were used as provided by the Genespring technology. Clustering for heatmaps was performed with Genespring GX 10 using centered Pearson's correlation metrics.

For determining the overlap to datasets from other platforms we used the EntrezGene IDs provided by Genespring technology, study author or Source tool (http://smd.stanford.edu/cgi-bin/source/sourceBatchSearch). The overlap to the dataset from Coller et. al. [23] was created using gene symbols. Fisher's exact test was used to prove statistical significance of the overlap.

**qRT-PCR**

RNA was isolated from the nine CAF strains incubated with or without Dasatinib used for microarray analysis. RNA samples were treated with DNase followed by cDNA synthesis using Revert Aid H Minus First strand cDNA synthesis Kit (Fermentas, St. Leon-Rot, Germany). Primers were taken from qPrimerDepot or designed with NCBI PrimerBlast for SVEP1. Platelet derived growth factor B (PDGFRB): sense AACTGTGCCCACACCAGAAG, antisense CAGGAGAGACAGCAACAGCA; sushi, von Willebrand factor type A, EGF and pentraxin domain containing 1 (SVEP1): sense GGCTGGCTGCTTGCCACAGT, antisense GAACCCCGGTGTGTGGAGCG; matrix metallopeptidase 1 (MMP1): sense TTCGGGGAGAAGTGATGTTC, antisense TTGTGGCCAGAAAACAGAAA; antigen identified by monoclonal antibody Ki-67 (MKI67): sense TGGGTCTGTTATTGATGAGCC, antisense TGACTTCCTTCCATTCTGAAGAC; TTK protein kinase (TTK): sense AGACCGTAGTGATAGCGACGGGG, antisense TCCATTTCTACAGAAAGCTGCGCTGG; forkhead box M1 (FOXM1): sense ATAGCAAGCGAGTCCGCATT, antisense TCTCCTCTTTCCCTGGTCCT; actin, beta (ACTB): sense GCACAGAGCCTCGCCTT, antisense GTTGTCGACGACGAGCG. Beta actin was used as an internal reference because it shows only minimal variability between different CAFs and upon Dasatinib treatment as seen with the microarrays. Each cDNA was assayed in triplicate.
