# Supplementary material for: Dasatinib reverses Cancer-associated Fibroblasts (CAFs) from primary Lung Carcinomas to a Phenotype comparable to that of normal Fibroblasts
Source: Mol Cancer. 2010 Jun 27;9:168. doi: 10.1186/1476-4598-9-168 (PMC2907332; doi:10.1186/1476-4598-9-168)
Supplement: Additional file 2 — Table S1. Potent CAF inhibitors [file 1476-4598-9-168-S2.PDF]

Table S1. Potent CAF inhibitors

**Substance Calbiochem Inhibitor Select Library (order number)**	**Growth (% of untreated control)**		**Targets**
	strain 1	strain 2	
PDK1/Akt/Flt Dual Pathway Inhibitor (#521275)	0.6	0.0	PDK1/Akt and Flt/PIM signaling pathways
Staurosporine, Streptomyces sp. (#569397)	10.2	4.2	Broad spectrum, e.g. CaMK, MLCK, PKA, PKC, PKG
PDGF Receptor Tyrosine Kinase Inhibitor IV (#521233)	47.4	43.0	PDGFR, c-Abl
K-252a, Nocardiopsis sp. (#420298)	47.5	43.4	Broad spectrum, e.g. CaMKII, MLCK, PKA, PKC
PI-103 (#528100)	49.9	31.7	DNA-PK, PI3-K, mTOR
