# Supplementary material for: Dasatinib reverses Cancer-associated Fibroblasts (CAFs) from primary Lung Carcinomas to a Phenotype comparable to that of normal Fibroblasts
Source: Mol Cancer. 2010 Jun 27;9:168. doi: 10.1186/1476-4598-9-168 (PMC2907332; doi:10.1186/1476-4598-9-168)
Supplement: Additional file 3 — Table S2. 492 genes regulated by Dasatinib [file 1476-4598-9-168-S3.PDF]

Table S2. 492 genes regulated by Dasatinib

Fold change (Dasatinib vs control)	gene description	genesymbol	GO biological process
**Genes downregulated by Dasatinib**			
2.52	arylacetamide deacetylase-like 1	AADACL1	metabolic process
2.15	adenylate kinase 5	AK5	dADP biosynthetic process, ATP metabolic process
3.19	anillin, actin binding protein	ANLN	cytokinesis, septin ring assembly, regulation of exit from mitosis
2.29	aldehyde oxidase 1	AOX1	oxygen and reactive oxygen species metabolic process, inflammatory response
3.54	apolipoprotein B mRNA editing enzyme, catalytic polypeptide-like 3B	APOBEC3B	no biological data available
2.80	Rho GTPase activating protein 11A	ARHGAP11A	signal transduction
2.44	Rho GTPase activating protein 18	ARHGAP18	signal transduction
2.00	Rho GTPase activating protein 19 | slit homolog 1 (Drosophila)	ARHGAP19|SLIT1	signal transduction
2.54	Rho GTPase activating protein 22	ARHGAP22	angiogenesis, regulation of transcription, DNA-dependent, signal transduction, cell differentiation
2.02	aryl hydrocarbon receptor nuclear translocator-like 2	ARNTL2	regulation of transcription, DNA-dependent, signal transduction, entrainment of circadian clock
2.69	ASF1 anti-silencing function 1 homolog B (S. cerevisiae)	ASF1B	chromatin assembly or disassembly, nucleosome assembly, regulation of transcription, DNA-dependent, cell differentiation
3.33	asp (abnormal spindle) homolog, microcephaly associated (Drosophila)	ASPM	mitosis
2.64	ATPase family, AAA domain containing 2	ATAD2	
2.36	ATPase family, AAA domain containing 5	ATAD5	response to DNA damage stimulus
3.02	aurora kinase A	AURKA	protein amino acid phosphorylation, spindle organization and biogenesis, mitosis, regulation of protein stability, phosphoinositide-mediated signaling
2.37	aurora kinase B	AURKB	cytokinesis, protein amino acid phosphorylation
2.36	baculoviral IAP repeat-containing 5 (survivin)	BIRC5	G2/M transition of mitotic cell cycle, anti-apoptosis, protein complex localization, spindle checkpoint,

2.69	breast cancer 1, early onset	BRCA1	cell cycle checkpoint, double-strand break repair via homologous recombination, signal transduction by p53 class mediator resulting in transcription of p21 class mediator and induction of apoptosis, negative regulation of cell cycle
2.46	breast cancer 2, early onset	BRCA2	double-strand break repair via homologous recombination, nucleotide-excision repair, regulation of transcription, DNA-dependent, DNA damage response, signal transduction by p53 class mediator resulting in transcription of p21 class mediator and induction of apoptosis
3.45		BUB1	protein amino acid phosphorylation, mitotic cell cycle spindle assembly checkpoint
3.24	BUB1 budding uninhibited by benzimidazoles 1 homolog beta (yeast)	BUB1B	protein amino acid phosphorylation, apoptosis, mitotic cell cycle checkpoint
2.20	chromosome 11 open reading frame 82	C11orf82	apoptosis, cell cycle arrest
2.14	chromosome 12 open reading frame 48	C12orf48	
2.09	chromosome 13 open reading frame 27	C13orf27	
3.35	chromosome 13 open reading frame 3	C13orf3	
2.36	chromosome 13 open reading frame 34	C13orf34	mitosis
2.07	chromosome 14 open reading frame 106	C14orf106	
2.32	chromosome 14 open reading frame 145	C14orf145	
2.60	chromosome 15 open reading frame 23	C15orf23	
2.30	chromosome 15 open reading frame 42	C15orf42	
2.74	chromosome 18 open reading frame 24	C18orf24	mitosis
2.40	chromosome 18 open reading frame 54	C18orf54	
2.01	chromosome 1 open reading frame 112	C1orf112	
2.01	chromosome 20 open reading frame 72	C20orf72	
2.36	chromosome 21 open reading frame 45	C21orf45	no biological data available
2.30	chromosome 6 open reading frame 167	C6orf167	
2.94	chromosome 6 open reading frame 173	C6orf173	

3.10		CD274	immune response, cell surface receptor linked signal transduction, negative regulation of T cell proliferation
3.22	cell division cycle 2, G1 to S and G2 to M	CDC2	protein amino acid phosphorylation, anti-apoptosis, traversing start control point of mitotic cell cycle
3.29	cell division cycle 20 homolog (S. cerevisiae)	CDC20	ubiquitin cycle, cell cycle
2.28	cell division cycle 25 homolog A (S. pombe)	CDC25A	regulation of cyclin-dependent protein kinase activity, protein amino acid dephosphorylation
2.35	cell division cycle 25 homolog C (S. pombe)	CDC25C	regulation of cyclin-dependent protein kinase activity, M phase of mitotic cell cycle, protein amino acid dephosphorylation, traversing start control point of mitotic cell cycle
3.00	CDC45 cell division cycle 45-like (S. cerevisiae)	CDC45L	DNA replication checkpoint, DNA replication initiation
3.48	cell division cycle 6 homolog (S. cerevisiae)	CDC6	DNA replication checkpoint, negative regulation of DNA replication, regulation of cyclin-dependent protein kinase activity, traversing start control point of mitotic cell cycle
2.14	cell division cycle 7 homolog (S. cerevisiae)	CDC7	G1/S transition of mitotic cell cycle, protein amino acid phosphorylation, positive regulation of cell proliferation, regulation of S phase
3.27	cell division cycle associated 2	CDCA2	mitosis
2.56	cell division cycle associated 3	CDCA3	ubiquitin cycle, mitosis
2.57	cell division cycle associated 5	CDCA5	G1/S transition of mitotic cell cycle, mitotic chromosome condensation, mitotic metaphase plate congression
2.73	cell division cycle associated 8	CDCA8	mitosis
2.26	cyclin-dependent kinase 2	CDK2	G2/M transition of mitotic cell cycle, protein amino acid phosphorylation, traversing start control point of mitotic cell cycle
3.96	cyclin-dependent kinase inhibitor 3 (CDK2-associated dual specificity phosphatase)	CDKN3	regulation of cyclin-dependent protein kinase activity, G1/S transition of mitotic cell cycle, cell cycle arrest, dephosphorylation
2.66	centromere protein A	CENPA	nucleosome assembly
3.07	centromere protein E, 312kDa	CENPE	mitotic chromosome movement towards spindle pole, kinetochore assembly
3.25	centromere protein F, 350/400ka (mitosin)	CENPF	mitotic cell cycle spindle assembly checkpoint, negative regulation of transcription, kinetochore assembly
2.10	centromere protein H	CENPH	kinetochore organization and biogenesis
3.62	centromere protein I	CENPI	sex differentiation
2.99	centromere protein K	CENPK	
2.37	centromere protein L	CENPL	
2.50	centromere protein N	CENPN	
2.52	centromere protein O	CENPO	
2.01	centrosomal protein 152kDa	CEP152	

3.20	cytoskeleton associated protein 2-like	CKAP2L	
2.98	CDC28 protein kinase regulatory subunit 1B	CKS1B	regulation of cyclin-dependent protein kinase activity
3.10	CDC28 protein kinase regulatory subunit 2	CKS2	regulation of cyclin-dependent protein kinase activity, spindle organization and biogenesis, phosphoinositide-mediated signaling
2.87	claspin homolog (Xenopus laevis)	CLSPN	DNA repair, cell cycle
2.24	carboxypeptidase A4	CPA4	proteolysis, histone acetylation
2.01	discoidin, CUB and LCCL domain containing 2	DCBLD2	cell adhesion, negative regulation of cell growth, intracellular receptor-mediated signaling pathway, wound healing
2.07	DEAD (Asp-Glu-Ala-Asp) box polypeptide 39	DDX39	nuclear mRNA splicing, via spliceosome, mRNA export from nucleus
3.67	DEP domain containing 1	DEPDC1	intracellular signaling cascade
2.70	DEP domain containing 1B	DEPDC1B	intracellular signaling cascade
2.45	dihydrofolate reductase	DHFR	glycine biosynthetic process, nucleotide biosynthetic process
2.18	diaphanous homolog 3 (Drosophila)	DIAPH3	actin cytoskeleton organization and biogenesis
2.20	dickkopf homolog 1 (Xenopus laevis)	DKK1	multicellular organismal development, negative regulation of Wnt receptor signaling pathway
2.52	deleted in lymphocytic leukemia, 2	DLEU2	
3.67	discs, large homolog 7 (Drosophila)	DLG7	M phase of mitotic cell cycle, mitotic chromosome movement towards spindle pole, cell-cell signaling, cell proliferation
2.47	DNA replication helicase 2 homolog (yeast)	DNA2	DNA replication
2.32		DSN1	chromosome segregation, mitosis
3.45	denticleless homolog (Drosophila)	DTL	DNA replication, ubiquitin cycle, response to DNA damage stimulus
2.06	E2F transcription factor 7	E2F7	regulation of transcription, DNA-dependent, cell cycle
2.71	epithelial cell transforming sequence 2 oncogene	ECT2	regulation of Rho protein signal transduction, positive regulation of I-kappaB kinase/NF-kappaB cascade
2.07	ELOVL family member 6, elongation of long chain fatty acids (FEN1/Elo2, SUR4/Elo3-like, yeast)	ELOVL6	fatty acid elongation
2.66	ectonucleotide pyrophosphatase/ phosphodiesterase 1	ENPP1	negative regulation of cell growth, cellular phosphate ion homeostasis, sequestering of triacylglycerol, negative regulation of protein amino acid autophosphorylation, negative regulation of insulin receptor signaling pathway, 3'-phosphoadenosine 5'-phosphosulfate metabolic

2.61	exonuclease 1	EXO1	mismatch repair, DNA recombination, immune response
2.43	enhancer of zeste homolog 2 (Drosophila)	EZH2	establishment and/or maintenance of chromatin architecture, regulation of transcription, DNA-dependent
2.27	fatty acid binding protein 5 (psoriasis-associated) | fatty acid binding protein 5-like 7	FABP5|FABP5L7	lipid metabolic process, transport, epidermis development
3.44	family with sequence similarity 111, member B	FAM111B	
2.59	family with sequence similarity 54, member A	FAM54A	
2.04	family with sequence similarity 64, member A	FAM64A	
2.92	family with sequence similarity 72, member A/B/D | gastric cancer up-regulated-2	FAM72A|FAM72B|GCUD2|FAM72D	
2.07	Fanconi anemia, complementation group A	FANCA	DNA repair, protein complex assembly
2.81	Fanconi anemia, complementation group D2	FANCD2	DNA repair, cell cycle, response to gamma radiation
3.36	Fanconi anemia, complementation group I	FANCI	DNA repair, cell cycle
2.00	Fanconi anemia, complementation group M	FANCM	DNA repair
2.65	F-box protein 5	FBXO5	cell division, negative regulation of ubiquitin-protein ligase activity during mitotic cell cycle
2.56	flap structure-specific endonuclease 1	FEN1	DNA replication, double-strand break repair, UV protection, phosphoinositide-mediated signaling
2.26	fibroblast growth factor 5	FGF5	cell-cell signaling, cell proliferation, fibroblast growth factor receptor signaling pathway
2.09	fidgetin-like 1	FIGNL1	ATP metabolic process
2.06		FLJ42986	regulation of transcription, DNA-dependent
2.89	forkhead box M1	FOXM1	regulation of transcription, DNA-dependent
2.10	G0/G1switch 2	G0S2	cell cycle
2.60	growth arrest-specific 2 like 3	GAS2L3	cell cycle arrest
3.10	gastric cancer up-regulated-2 | family with sequence similarity 72, member D/A	GCUD2|FAM72D|FAM72A	
2.06	Gen homolog 1, endonuclease (Drosophila)	GEN1	DNA repair
2.80	GINS complex subunit 1 (Psf1 homolog)	GINS1	inner cell mass cell proliferation, DNA replication
2.29	GINS complex subunit 2 (Psf2 homolog)	GINS2	DNA replication
2.63	GINS complex subunit 4 (Sld5 homolog)	GINS4	DNA replication

2.47	H2A histone family, member Z	H2AFZ	nucleosome assembly
2.42	hyaluronan synthase 2	HAS2	
2.01	heparin-binding EGF-like growth factor	HBEGF	epidermal growth factor receptor signaling pathway, wound healing, spreading of epidermal cells, positive regulation of smooth muscle cell proliferation and keratinocyte migration
2.90	helicase, lymphoid-specific	HELLS	mitosis, maintenance of DNA methylation, centric heterochromatin formation
2.12	hect domain and RLD 4	HERC4	ubiquitin cycle
2.44	histone cluster 1, H1a	HIST1H1A	nucleosome assembly
2.93	histone cluster 1, H1b	HIST1H1B	nucleosome assembly
2.08	histone cluster 1, H1e	HIST1H1E	nucleosome assembly, nucleosome positioning
2.99	histone cluster 1, H2ab/H2ae	HIST1H2AB|HIST1H2AE	nucleosome assembly
2.02	histone cluster 1, H2ag/H2ai/H2ak/H2aj/H2al/H2am	HIST1H2AG |and others	nucleosome assembly
2.21	histone cluster 1, H2ai/H2ak/H2aj/H2al/H2am/H2ag/H3f	HIST1H2AI| and others	nucleosome assembly
2.20	histone cluster 1, H2ai/H3h/H2ak/H2aj/H2al/H2am/H2ag/H2bn/H3a/H3d/H3c/H3e/H3i/H3g/H3j/H3b/H3f	HIST1H2AI| and others	nucleosome assembly
2.22	histone cluster 1, H2ak/H2ai/H2aj/H2al/H2am/H2ag	HIST1H2AK| and others	nucleosome assembly
2.07	histone cluster 1, H2bb	HIST1H2BB	nucleosome assembly
2.59	histone cluster 1, H2bf/H2bg/H2be/H2bi/ H2bc	HIST1H2BF| and others	nucleosome assembly, defense response to bacterium
2.17	histone cluster 1, H2bh	HIST1H2BH	nucleosome assembly
2.08	histone cluster 1, H2bk | H2B histone family, member S	HIST1H2BK| H2BFS	nucleosome assembly, defense response to bacterium
3.15	histone cluster 1, H2bm	HIST1H2BM	nucleosome assembly
2.55	histone cluster 1, H3a/H2bn/H3d/H3c/H3e/H3i/H3g/H3j/H3h/H3b/ H3f	HIST1H3A| and others	nucleosome assembly
3.14	histone cluster 1, H3b/H2bn/H3a/H3d/H3c/H3e/H3i/H3g/H3j/H3h/H3f	HIST1H3B| and others	nucleosome assembly
2.43	histone cluster 1, H3d/H2ad/H2bn/H3a/H3c/H3e/H3i/H3g/H3j/H3h/H3b/H3f	HIST1H3D| and others	nucleosome assembly
2.58	histone cluster 1, H3f/H2bn/H3a/H3d/H3c/H3e/H3i/H3g/H3j/H3h/H3b	HIST1H3F| and others	nucleosome assembly

2.68	histone cluster 1, H4b /H4i/H4a/H4d/H4f/H4k/H4j/H4c/H4 h/H4e/H4l | histone cluster 4, H4 | histone cluster 2, H4b/H4a	HIST1H4B| and others	establishment and/or maintenance of chromatin architecture, nucleosome assembly, phosphoinositide-mediated signaling
2.05	histone cluster 1, H4c/H4i/H4a/H4d/H4f /H4k/H4j/H4h/H4b/H4e/H4l | histone cluster 4, H4 | histone cluster 2, H4b/H4a	HIST1H4C| and others	establishment and/or maintenance of chromatin architecture, nucleosome assembly, phosphoinositide-mediated signaling
2.90	histone cluster 1, H4d/H4i/H4a/H4f/H4k/H4j/H4c/H4 h/H4b/H4e/H4l | histone cluster 4, H4 | histone cluster 2 H4a	HIST1H4D| and others	establishment and/or maintenance of chromatin architecture, nucleosome assembly, phosphoinositide-mediated signaling
2.03	histone cluster 1, H4k/H4j/H4i/H4a/H4d/H4f/H4c/ H4h/H4b/H4e/H4l | histone cluster 4, H4 | histone cluster 2, H4b/H4a	HIST1H4K| and others	establishment and/or maintenance of chromatin architecture, nucleosome assembly, phosphoinositide-mediated signaling
2.65	histone cluster 1, H4l/H4i/H4a/H4d/H4f/H4k/H4j/ H4c/H4h/H4b/H4e | histone cluster 4, H4 | histone cluster 2, H4b/H4a	HIST1H4L| and others	establishment and/or maintenance of chromatin architecture, nucleosome assembly, phosphoinositide-mediated signaling
2.53	histone cluster 2, H2ab	HIST2H2AB	nucleosome assembly
2.58	histone cluster 2, H3d/H3c/H3a	HIST2H3D| HIST2H3C| HIST2H3A	nucleosome assembly
2.67	Holliday junction recognition protein	HJURP	
2.65	high mobility group AT-hook 2	HMGA2	regulation of cell growth, establishment and/or maintenance of chromatin architecture, regulation of transcription, DNA-dependent, cell division
2.31	high-mobility group box 2	HMGB2	DNA unwinding during replication, base-excision repair, DNA ligation, nucleosome assembly, phosphoinositide-mediated signaling
3.44	hyaluronan-mediated motility receptor (RHAMM)	HMMR	cell motility
2.57	IQ motif containing GTPase activating protein 3	IQGAP3	regulation of small GTPase mediated signal transduction
3.26	integrin, alpha 6	ITGA6	cell-substrate junction assembly, cell-matrix adhesion, integrin-mediated signaling pathway
2.03	integrin beta 3 binding protein (beta3-endonexin)	ITGB3BP	regulation of transcription, DNA-dependent, apoptosis, cell adhesion, signal transduction
2.49	potassium channel, subfamily K, member 2	KCNK2	potassium ion transport, G-protein coupled receptor protein signaling pathway
3.71		KIAA0101	
3.25		KIAA1524	

2.35	kinesin family member 23	KIF23	mitotic spindle elongation, microtubule-based movement, cell division
3.14	kinesin family member 2C	KIF2C	microtubule-based movement, mitosis, establishment and/or maintenance of microtubule cytoskeleton polarity
3.13	kinesin family member 4A	KIF4A	organelle organization and biogenesis, microtubule-based movement, anterograde axon cargo transport
3.10	kinesin family member C1	KIFC1	mitotic sister chromatid segregation, microtubule-based movement
2.57	kinetochore associated 1	KNTC1	protein complex assembly, mitotic cell cycle checkpoint, regulation of exit from mitosis
2.13	karyopherin alpha 2 (RAG cohort 1, importin alpha 1) | karyopherin alpha-2 subunit like	KPNA2|LOC728860	regulation of DNA recombination, M phase specific microtubule process, G2 phase of mitotic cell cycle, NLS-bearing substrate import into nucleus
2.05	keratin associated protein 2-4 | keratin associated protein 2-1 | hypothetical LOC644350 | similar to keratin associated protein | hypothetical LOC728934 | similar to keratin associated protein 2-4 | keratin associated protein 2.1B | hypothetical LOC728943 | keratin associated protein 2-2	KRTAP2-4|KRTAP2-1|LOC644350|LOC728285|LOC728934|LOC730755|KAP2.1B|LOC728943|KRTAP2-2	
2.94	lamin B1	LMNB1	
2.38		LOC100129478	
2.86	dihydrofolate reductase pseudogene	LOC1720	
2.17	ribosomal protein SA pseudogene	LOC204010	translation
2.10	similar to hect domain and RLD 2	LOC390561	
2.22	RNA, small nucleolar	LOC85391	
2.45	leupaxin	LPXN	protein complex assembly, cell adhesion, signal transduction
3.36	MAD2 mitotic arrest deficient-like 1 (yeast)	MAD2L1	mitotic cell cycle checkpoint, cell division
2.05	mal, T-cell differentiation protein-like	MALL	cholesterol homeostasis
2.31	membrane-associated ring finger (C3HC4) 3	MARCH3	ubiquitin cycle, endocytosis
2.07	microtubule associated serine/threonine kinase-like	MASTL	protein amino acid phosphorylation
2.90	minichromosome maintenance complex component 10	MCM10	DNA replication

2.27	minichromosome maintenance complex component 6	MCM6	DNA unwinding during replication, regulation of transcription, DNA-dependent
2.50	minichromosome maintenance complex component 7	MCM7	DNA replication initiation, regulation of transcription, DNA-dependent, response to DNA damage stimulus, regulation of phosphorylation
3.31	minichromosome maintenance complex component 8	MCM8	DNA replication initiation, regulation of transcription, DNA-dependent
3.06	maternal embryonic leucine zipper kinase	MELK	protein amino acid phosphorylation
2.14	similar to Six transmembrane epithelial antigen of prostate	MGC87042	
3.48	antigen identified by monoclonal antibody Ki-67	MKI67	cell proliferation
2.72	MLF1 interacting protein	MLF1IP	regulation of transcription, DNA-dependent
3.59	matrix metallopeptidase 1 (interstitial collagenase)	MMP1	proteolysis, collagen catabolic process
2.20	meiotic nuclear divisions 1 homolog (S. cerevisiae)	MND1	DNA recombination, meiosis
3.08	M-phase phosphoprotein 1	MPHOSPH1	microtubule-based movement, cell cycle arrest, regulation of mitosis
2.32	mutS homolog 2, colon cancer, nonpolyposis type 1 (E. coli)	MSH2	base-excision repair, mismatch repair, postreplication repair, maintenance of DNA repeat elements, negative regulation of cell cycle
2.20	Mdm2, transformed 3T3 cell double minute 2, p53 binding protein (mouse) binding protein, 104kDa	MTBP	ubiquitin cycle, cell cycle arrest
2.02	v-myb myeloblastosis viral oncogene homolog (avian)-like 1	MYBL1	regulation of transcription, DNA-dependent
2.15	v-myb myeloblastosis viral oncogene homolog (avian)-like 2	MYBL2	regulation of transcription, DNA-dependent, transcription from RNA polymerase II promoter, anti-apoptosis
2.46	myopalladin	MYPN	
2.13	non-SMC condensin I complex, subunit D2	NCAPD2	cell cycle, mitotic chromosome condensation
2.30	non-SMC condensin II complex, subunit D3	NCAPD3	mitotic chromosome condensation, cell division
3.37	non-SMC condensin I complex, subunit G	NCAPG	mitotic chromosome condensation, cell division
2.67	non-SMC condensin II complex, subunit G2	NCAPG2	mitotic chromosome condensation
3.11	non-SMC condensin I complex, subunit H	NCAPH	mitotic chromosome condensation, cell division
3.26		NDC80	mitotic sister chromatid segregation, spindle organization and biogenesis, phosphoinositide-mediated signaling
2.41	neurofilament, medium polypeptide 150kDa	NEFM	microtubule cytoskeleton organization and biogenesis, axon cargo transport, regulation of axon

3.26	nucleolar and spindle associated protein 1	NUSAP1	cytokinesis after mitosis, mitotic chromosome condensation, establishment of mitotic spindle localization, cell division
2.54	origin recognition complex, subunit 1-like (yeast)	ORC1L	DNA replication initiation
2.69	origin recognition complex, subunit 6 like (yeast)	ORC6L	DNA replication
4.39	PDZ binding kinase	PBK	protein amino acid phosphorylation, mitosis
2.05	plasminogen activator, urokinase	PLAU	proteolysis, chemotaxis, signal transduction, fibrinolysis
3.16	polo-like kinase 1 (Drosophila)	PLK1	protein amino acid phosphorylation, mitosis, cell proliferation, cell division
3.46	polo-like kinase 4 (Drosophila)	PLK4	protein amino acid phosphorylation
2.17	polymerase (DNA directed), alpha 1	POLA1	S phase of mitotic cell cycle, DNA synthesis during DNA repair, DNA replication initiation, leading strand elongation, lagging strand elongation , double-strand break repair via nonhomologous end joining, cell proliferation
2.51	polymerase (DNA directed), alpha 2 (70kD subunit)	POLA2	protein import into nucleus, translocation, DNA replication
2.63	polymerase (DNA directed), epsilon 2 (p59 subunit)	POLE2	DNA replication, DNA repair
2.34	polymerase (DNA directed), theta	POLQ	DNA replication, DNA repair
2.77	polymerase (RNA) III (DNA directed) polypeptide G (32kD)	POLR3G	regulation of transcription from RNA polymerase III promoter
2.21	peptidylprolyl isomerase (cyclophilin)-like 5	PPIL5	ubiquitin cycle
3.09	protein regulator of cytokinesis 1	PRC1	mitotic spindle elongation, cytokinesis
2.73	primase, DNA, polypeptide 1 (49kDa)	PRIM1	DNA replication, synthesis of RNA primer, transcription
2.28	primase, DNA, polypeptide 2 (58kDa)	PRIM2	DNA replication, synthesis of RNA primer, transcription
3.07	proline rich 11	PRR11	
2.52	PSMC3 interacting protein	PSMC3IP	DNA recombination, meiosis
2.84	pituitary tumor-transforming 1	PTTG1	DNA metabolic process, DNA repair, transcription from RNA polymerase II promoter, chromosome segregation, chromosome organization and biogenesis, cell division
3.22	pentraxin-related gene, rapidly induced by IL-1 beta	PTX3	inflammatory response, opsonization, positive regulation of nitric oxide biosynthetic process, positive regulation of phagocytosis
2.61	Rac GTPase activating protein 1	RACGAP1	cytokinesis, contractile ring formation, ion transport, cell cycle, intracellular signaling cascade, neuroblast proliferation, sulfate transport, cell differentiation
2.13		RAD51	double-strand break repair via homologous

2.10	replication factor C (activator 1) 2, 40kDa	RFC2	DNA replication
2.78	replication factor C (activator 1) 3, 38kDa	RFC3	DNA synthesis during DNA repair, DNA strand elongation during DNA replication
2.34	replication factor C (activator 1) 4, 37kDa	RFC4	DNA strand elongation during DNA replication, phosphoinositide-mediated signaling
2.15	replication factor C (activator 1) 5, 36.5kDa	RFC5	DNA replication, DNA repair
2.12	ring finger and WD repeat domain 3	RFWD3	
2.59	RGM domain family, member B	RGMB	cell adhesion, BMP signaling pathway, positive regulation of transcription
2.20	regulator of G-protein signaling 4	RGS4	inactivation of MAPK activity, regulation of G-protein coupled receptor protein signaling pathway, negative regulation of signal transduction
2.23	ribonuclease H2, subunit A	RNASEH2A	DNA replication, RNA catabolic process
2.76	RNA, U5F small nuclear	RNU5F	
2.22	ribonucleotide reductase M1	RRM1	DNA replication
3.23	ribonucleotide reductase M2 polypeptide	RRM2	DNA replication, deoxyribonucleoside diphosphate metabolic process
2.49	sema domain, immunoglobulin domain (Ig), short basic domain, secreted, (semaphorin) 3A	SEMA3A	multicellular organismal development, cell differentiation
3.63	serpin peptidase inhibitor, clade B (ovalbumin), member 2	SERPINB2	anti-apoptosis
2.07	serpin peptidase inhibitor, clade B (ovalbumin), member 7	SERPINB7	
2.79	shugoshin-like 1 (S. pombe)	SGOL1	mitosis, meiotic chromosome segregation
2.75	shugoshin-like 2 (S. pombe)	SGOL2	chromosome segregation, cell division
3.57	SHC SH2-domain binding protein 1	SHCBP1	
2.07	solute carrier family 16, member 6 (monocarboxylic acid transporter 7)	SLC16A6	monocarboxylic acid transport
2.03	solute carrier family 20 (phosphate transporter), member 1	SLC20A1	phosphate metabolic process, phosphate transport, positive regulation of I-kappaB kinase/NF-kappaB cascade
2.11	schlafen family member 11	SLFN11	
2.65	structural maintenance of chromosomes 2	SMC2	DNA metabolic process, mitotic chromosome condensation, cell division
2.59	structural maintenance of chromosomes 4	SMC4	DNA metabolic process, mitotic chromosome condensation, cell division
2.83	small nucleolar RNA, C/D box 30	SNORD30	
2.11	small nucleolar RNA, C/D box 50B	SNORD50B	

2.04	sprouty homolog 2 (Drosophila)	SPRY2	cell-cell signaling, organ morphogenesis, lung development, negative regulation of MAP kinase activity, cell fate commitment
2.61	stanniocalcin 1	STC1	cellular calcium ion homeostasis, cell surface receptor linked signal transduction, cell-cell signaling, response to nutrient
2.31	six transmembrane epithelial antigen of the prostate 1	STEAP1	iron ion transport
2.53	SCL/TAL1 interrupting locus	STIL	multicellular organismal development, cell proliferation
2.22	stathmin 1/oncoprotein 18	STMN1	microtubule depolymerization, mitotic spindle organization and biogenesis, intracellular signaling cascade, multicellular organismal development, cell differentiation
2.89	transforming, acidic coiled-coil containing protein 3	TACC3	
3.02	transcription factor 19 (SC1)	TCF19	regulation of transcription from RNA polymerase II promoter, cell proliferation
2.14	tyrosyl-DNA phosphodiesterase 1	TDP1	DNA repair
2.36	timeless homolog (Drosophila)	TIMELESS	morphogenesis of an epithelium, response to DNA damage stimulus, circadian rhythm, detection of abiotic stimulus, negative regulation of transcription
2.19	TIMELESS interacting protein	TIPIN	DNA replication checkpoint, mitosis, positive regulation of cell proliferation, intra-S DNA damage checkpoint, replication fork protection
2.41	thymidine kinase 1, soluble	TK1	DNA replication
2.28	transmembrane protein 194	TMEM194	no biological data available
2.67	transmembrane protein 48	TMEM48	nuclear pore distribution, mRNA transport, nuclear pore complex assembly, intracellular protein transport across a membrane
2.45	thymopoietin	TMPO	regulation of transcription
2.90	topoisomerase (DNA) II alpha 170kDa	TOP2A	DNA replication, DNA ligation, DNA repair, chromosome segregation, apoptotic chromosome condensation, positive regulation of apoptosis, phosphoinositide-mediated signaling
3.17		TPX2	mitosis, cell proliferation
2.92	thyroid hormone receptor interactor 13	TRIP13	transcription from RNA polymerase II promoter
2.08	transient receptor potential cation channel, subfamily A, member 1	TRPA1	ion transport, response to cold
2.02	trypsinogen C | protease, serine, 2 (trypsin 2) | protease, serine, 1 (trypsin 1) | protease, serine, 3 | hypothetical protein LOC100134294	TRY6|PRSS2|PRSS1|PRSS3|LOC100134294	proteolysis, digestion, positive regulation of cell growth, collagen catabolic process, positive regulation of cell adhesion

2.12	ubiquitin-conjugating enzyme E2S	UBE2S	ubiquitin cycle, regulation of protein metabolic process
2.77	ubiquitin-conjugating enzyme E2T (putative)	UBE2T	ubiquitin cycle, regulation of protein metabolic process
2.32	ubiquitin-like, containing PHD and RING finger domains, 1	UHRF1	DNA repair, regulation of transcription from RNA polymerase II promoter, ubiquitin cycle, cell cycle, cell proliferation
2.02	HWKM1940	UNQ1940	
2.60	ventricular zone expressed PH domain homolog 1 (zebrafish)	VEPH1	
2.65	vaccinia related kinase 1	VRK1	protein amino acid phosphorylation
2.44	WD repeat and HMG-box DNA binding protein 1	WDHD1	regulation of transcription, DNA-dependent
2.71	WD repeat domain 76	WDR76	
2.06	Wolf-Hirschhorn syndrome candidate 1	WHSC1	regulation of transcription, DNA-dependent, chromatin modification
3.02	X-ray repair complementing defective repair in Chinese hamster cells 2	XRCC2	DNA repair, DNA recombination
2.69	Zwilch, kinetochore associated, homolog (Drosophila)	ZWILCH	mitotic cell cycle checkpoint, cell division
3.13	ZW10 interactor	ZWINT	mitotic sister chromatid segregation, spindle organization and biogenesis, mitotic cell cycle checkpoint, phosphoinositide-mediated signaling, establishment of localization in cell

Fold change (Dasatinib vs control)	gene description	genesymbol	GO biological process
**Genes upregulated by Dasatinib**			
2.61	alpha-2-macroglobulin	A2M	intracellular protein transport, response to carbon dioxide and to glucocorticoid stimulus
2.11	ATP-binding cassette, sub-family A (ABC1), member 1	ABCA1	lysosome organization and biogenesis, intracellular cholesterol transport, cholesterol efflux
2.25	ATP-binding cassette, sub-family C (CFTR/MRP), member 3	ABCC3	transport
2.56	abhydrolase domain containing 4	ABHD4	proteolysis, lipid catabolic process
2.53	aggrecan	ACAN	cell adhesion, skeletal development, proteolysis
3.64	actin, alpha 2, smooth muscle, aorta	ACTA2	
8.26	actin, alpha, cardiac muscle 1	ACTC1	muscle thin filament assembly, apoptosis
3.82	actin, gamma 2, smooth muscle, enteric	ACTG2	
2.24	ADAM metallopeptidase with thrombospondin type 1 motif, 5 (aggrecanase-2)	ADAMTS5	proteolysis
2.05	aldo-keto reductase family 1, member C3 (3-alpha hydroxysteroid dehydrogenase, type II)	AKR1C3	prostaglandin metabolic process
6.83	aldehyde dehydrogenase 1 family, member A1	ALDH1A1	aldehyde metabolic process
2.15	aldehyde dehydrogenase 1 family, member L2	ALDH1L2	one-carbon compound metabolic process, 10-formyltetrahydrofolate catabolic process
2.79	aldehyde dehydrogenase 2 family (mitochondrial)	ALDH2	carbohydrate metabolic process, alcohol metabolic process
2.00	alpha-kinase 2	ALPK2	protein amino acid phosphorylation
2.72	adhesion molecule with Ig-like domain 2	AMIGO2	homophilic cell adhesion, heterophilic cell adhesion, negative regulation of programmed cell death
2.23	angiopoietin-like 2	ANGPTL2	signal transduction, multicellular organismal development
2.61	ankylosis, progressive homolog (mouse)	ANKH	phosphate transport, regulation of bone mineralization
2.22	anthrax toxin receptor 1	ANTXR1	
2.06	armadillo repeat containing 9	ARMC9	
2.59	asparagine synthetase	ASNS	negative regulation of apoptosis, asparagine biosynthetic process, cellular response to glucose

2.05	biglycan	BGN	no biological data available
2.45	chromosome 10 open reading frame 10	C10orf10	
4.30	chromosome 1 open reading frame 198	C1orf198	
3.97	complement component 1, r subcomponent	C1R	complement activation, classical pathway
4.25	complement component 1, s subcomponent	C1S	complement activation, classical pathway, G-protein coupled receptor protein signaling pathway
2.07	chromosome 4 open reading frame 18	C4orf18	
2.02	chromosome 5 open reading frame 13	C5orf13	regulation of transforming growth factor beta receptor signaling pathway
2.02	chromosome 9 open reading frame 19	C9orf19	
2.59	chromosome 9 open reading frame 3	C9orf3	proteolysis, leukotriene biosynthetic process
2.37	calcium channel, voltage-dependent, L type, alpha 1C subunit	CACNA1C	elevation of cytosolic calcium ion concentration
2.00	calcium binding and coiled-coil domain 1	CALCOCO1	transcription, Wnt receptor signaling pathway, steroid hormone receptor signaling pathway
2.16		CD302	
3.87	carboxylesterase 1 (monocyte/macrophage serine esterase 1)	CES1	metabolic process, response to toxin
2.15	ChaC, cation transport regulator homolog 1 (E. coli)	CHAC1	
2.42	chitinase 3-like 1 (cartilage glycoprotein-39)	CHI3L1	carbohydrate metabolic process
2.24	clusterin	CLU	lipid metabolic process, apoptosis, anti-apoptosis, complement activation, classical pathway
2.57	calponin 1, basic, smooth muscle	CNN1	regulation of smooth muscle contraction, actomyosin structure organization and biogenesis
2.17	cyclin M2	CNNM2	ion transport
2.54	collagen, type XI, alpha 1	COL11A1	cartilage condensation, phosphate transport, extracellular matrix organization and biogenesis
2.33	collagen, type III, alpha 1 (Ehlers-Danlos syndrome type IV, autosomal dominant)	COL3A1	phosphate transport, cell-matrix adhesion, transforming growth factor beta receptor signaling pathway, integrin-mediated signaling pathway, collagen fibril organization, response to cytokine stimulus, wound healing
3.56	collagen, type IV, alpha 1	COL4A1	phosphate transport
2.49	collagen, type IV, alpha 2	COL4A2	phosphate transport, negative regulation of angiogenesis, extracellular matrix organization and

2.79	cysteine-rich secretory protein LCCL domain containing 2	CRISPLD2	lung development
2.35	crystallin, alpha B	CRYAB	protein folding, anti-apoptosis, transmembrane receptor protein tyrosine kinase signaling pathway
2.41	cathepsin D | similar to RIKEN cDNA 6330512M04 gene (mouse)	CTSD|LOC402778	proteolysis
2.40	cytochrome P450, family 1, subfamily B, polypeptide 1	CYP1B1	estrogen metabolic process
2.30	dapper, antagonist of beta-catenin, homolog 1 (Xenopus laevis)	DACT1	Wnt receptor signaling pathway
3.05	DNA-damage-inducible transcript 4	DDIT4	apoptosis, negative regulation of signal transduction
2.34	DEP domain containing 6	DEPDC6	intracellular signaling cascade
2.78	dehydrogenase/reductase (SDR family) member 3	DHRS3	retinol metabolic process
2.65	dipeptidyl-peptidase 4 (CD26, adenosine deaminase complexing protein 2)	DPP4	proteolysis, immune response
2.09	enoyl Coenzyme A hydratase 1, peroxisomal	ECH1	generation of precursor metabolites and energy, fatty acid beta-oxidation
2.38	EGF-like repeats and discoidin I-like domains 3	EDIL3	cell adhesion, multicellular organismal development
2.23	EGF-containing fibulin-like extracellular matrix protein 1	EFEMP1	visual perception
4.58	elastin (supravalvular aortic stenosis, Williams-Beuren syndrome)	ELN	DNA repair, respiratory gaseous exchange, blood circulation, organ morphogenesis
3.79	endothelial PAS domain protein 1	EPAS1	angiogenesis, response to hypoxia, regulation of transcription, DNA-dependent, signal transduction
2.31	epoxide hydrolase 1, microsomal (xenobiotic)	EPHX1	xenobiotic metabolic process, response to toxin, aromatic compound catabolic process
2.11	family with sequence similarity 150, member A	FAM150A	
3.12	fibulin 5	FBLN5	cell-matrix adhesion, blood coagulation
2.43	F-box protein 32	FBXO32	ubiquitin cycle
2.75	fibroblast growth factor 9 (glia-activating factor)	FGF9	angiogenesis, positive regulation of mesenchymal cell proliferation, protein import into nucleus, cell-cell signaling, negative regulation of Wnt receptor signaling pathway , lung development, positive regulation of VEGFR, FGFR and smoothened signaling pathway, regulation of timing of cell differentiation
3.53	fibrinogen-like 2	FGL2	signal transduction
2.71	four and a half LIM domains 1	FHL1	organ morphogenesis, cell growth, cell

2.79	glypican 4	GPC4	cell proliferation, anatomical structure morphogenesis
2.21	glycoprotein (transmembrane) nmb	GPNMB	negative regulation of cell proliferation
2.38	gremlin 2, cysteine knot superfamily, homolog (Xenopus laevis)	GREM2	
2.20	glutathione S-transferase M5	GSTM5	metabolic process
2.02	GTF2I repeat domain containing 2 / 2B	GTF2IRD2| GTF2IRD2B	regulation of transcription, DNA-dependent
2.64	hyaluronan and proteoglycan link protein 3	HAPLN3	cell adhesion
2.30	HEG homolog 1 (zebrafish)	HEG1	
2.83	hephaestin	HEPH	copper ion transport, iron ion transport
3.69	hemicentin 1	HMCN1	protein amino acid phosphorylation, cell adhesion, bioluminescence, protein-chromophore linkage, response to stimulus
2.10	5-hydroxytryptamine (serotonin) receptor 2B	HTR2B	G-protein signaling, coupled to IP3 second messenger (phospholipase C activating), blood circulation, positive regulation of I-kappaB kinase/NF-kappaB cascade
2.14	HtrA serine peptidase 1	HTRA1	regulation of cell growth, proteolysis, negative regulation of TGFβ and BMP signaling pathway
2.17	insulin-like growth factor 2 (somatomedin A)	IGF2	genetic imprinting, cell proliferation, insulin receptor signaling pathway
2.09	insulin-like growth factor binding protein 5	IGFBP5	regulation of cell growth, signal transduction
2.20	insulin-like growth factor binding protein 7	IGFBP7	regulation of cell growth, negative regulation of cell proliferation
2.80	interleukin 6 (interferon, beta 2)	IL6	neutrophil apoptosis, cell surface receptor linked signal transduction, negative regulation of apoptosis, positive regulation of MAPKKK cascade, negative regulation of chemokine biosynthetic process, positive regulation of T-helper 2 cell differentiation, negative regulation of hormone secretion, response to glucocorticoid stimulus
2.85	integrin, alpha 11	ITGA11	substrate-bound cell migration, cell-matrix adhesion, integrin-mediated signaling pathway
2.19	integrin, alpha V (vitronectin receptor, alpha polypeptide, antigen CD51)	ITGAV	blood vessel development, cell-matrix adhesion, integrin-mediated signaling pathway
2.18	inositol 1,4,5-triphosphate receptor, type 1	ITPR1	calcium ion transport, signal transduction
2.14	jagged 1 (Alagille syndrome)	JAG1	angiogenesis, morphogenesis of an epithelial sheet, cell communication, myoblast differentiation
2.04	junction-mediating and regulatory	JMY	DNA repair, regulation of transcription from RNA

2.20	laminin, beta 1	LAMB1	cell adhesion, positive regulation of cell migration, positive regulation of epithelial cell proliferation
3.75	limb bud and heart development homolog (mouse)	LBH	regulation of transcription, DNA-dependent, multicellular organismal development
2.57	LIM and calponin homology domains 1	LIMCH1	actomyosin structure organization and biogenesis
3.85	LIM and cysteine-rich domains 1	LMCD1	negative regulation of transcription from RNA polymerase II promoter
3.56	leiomodin 1 (smooth muscle)	LMOD1	
3.08		LOC728264	
2.48	loss of heterozygosity, 3, chromosomal region 2, gene A	LOH3CR2A	
2.05	low density lipoprotein-related protein 1 (alpha-2-macroglobulin receptor)	LRP1	lipid metabolic process, endocytosis, multicellular organismal development, cell proliferation
2.40	limbic system-associated membrane protein	LSAMP	cell adhesion
2.58	lumican	LUM	collagen fibril organization
2.45	MAM domain containing 2	MAMDC2	
2.48	melanoma cell adhesion molecule	MCAM	cell adhesion, anatomical structure morphogenesis
4.81	MAM domain containing glycosylphosphatidylinositol anchor 1	MDGA1	neuron migration, spinal cord association neuron differentiation
6.35	microfibrillar-associated protein 4	MFAP4	cell adhesion, signal transduction
2.15	milk fat globule-EGF factor 8 protein	MFGE8	cell adhesion, single fertilization
3.62	matrix Gla protein	MGP	cartilage condensation, ossification, response to mechanical stimulus, response to hormone stimulus, cell differentiation, lung development
3.07	murine retrovirus integration site 1 homolog	MRVI1	
2.64	myosin, heavy chain 10, non-muscle	MYH10	cytokinesis after mitosis, regulation of cell shape, actin filament-based movement
2.28	myosin, heavy chain 11, smooth muscle	MYH11	muscle contraction, muscle thick filament assembly, elastic fiber assembly
2.37	myosin light chain kinase	MYLK	protein amino acid phosphorylation
2.30	neogenin homolog 1 (chicken)	NEO1	cell motility, cell adhesion, cell-cell signaling, multicellular organismal development
2.19	nicotinamide N-methyltransferase	NNMT	
2.11	neuropilin 2	NRP2	angiogenesis, cell adhesion, axon guidance, cell differentiation, cell redox homeostasis
2.38	neurexin 3	NRXN3	cell adhesion, axon guidance
3.03	NUAK family, SNF1-like kinase, 1	NUAK1	protein amino acid phosphorylation

2.05	pre-B-cell leukemia homeobox 1	PBX1	regulation of transcription, DNA-dependent, transcription from RNA polymerase II promoter, C21-steroid hormone biosynthetic process, sex determination, cell differentiation
2.28	phosphoenolpyruvate carboxykinase 2 (mitochondrial)	PCK2	gluconeogenesis
2.58	protein-L-isoaspartate (D-aspartate) O-methyltransferase domain containing 1	PCMTD1	protein modification process
2.10	phosphodiesterase 3A, cGMP-inhibited	PDE3A	lipid metabolic process, signal transduction
2.55	platelet-derived growth factor receptor, beta polypeptide	PDGFRB	transmembrane receptor protein tyrosine kinase signaling pathway, regulation of peptidyl-tyrosine phosphorylation
2.06	pelota homolog (Drosophila) | integrin, alpha 1	PELO|ITGA1	translation, cell-matrix adhesion, integrin-mediated signaling pathway, neutrophil chemotaxis, cellular extravasation
2.05	plasma glutamate carboxypeptidase	PGCP	proteolysis
2.50	pleckstrin homology domain containing, family H (with MyTH4 domain) member 2	PLEKHH2	
2.09	phospholamban	PLN	cellular calcium ion homeostasis, negative regulation of heart contraction
2.62	phospholipid scramblase 4	PLSCR4	blood coagulation, phospholipid scrambling
4.63	plexin domain containing 2	PLXDC2	multicellular organismal development
5.25	podocan	PODN	
2.44	podocan-like 1	PODNL1	
2.74	protein phosphatase 1, regulatory (inhibitor) subunit 3C	PPP1R3C	carbohydrate metabolic process, glycogen biosynthetic process
2.43	phosphoserine aminotransferase 1	PSAT1	L-serine biosynthetic process, pyridoxine biosynthetic process
2.14	prostaglandin I2 (prostacyclin) synthase	PTGIS	prostaglandin biosynthetic process, lipid metabolic process, fatty acid biosynthetic process
2.56	Ras association (RalGDS/AF-6) domain family member 2	RASSF2	signal transduction, negative regulation of cell cycle
2.17	ribonucleoprotein, PTB-binding 2	RAVER2	
4.40	retinol dehydrogenase 10 (all-trans)	RDH10	visual perception, metabolic process
2.02	reversion-inducing-cysteine-rich protein with kazal motifs	RECK	negative regulation of cell cycle
2.15	receptor tyrosine kinase-like orphan receptor 1	ROR1	protein amino acid phosphorylation, transmembrane receptor protein tyrosine kinase signaling pathway
2.17	syndecan 2	SDC2	no biological data available
2.10	selenoprotein P, plasma, 1	SEPP1	selenium metabolic process, response to oxidative stress, brain development, growth

2.53	solute carrier family 1 (glutamate/neutral amino acid transporter), member 4	SLC1A4	dicarboxylic acid transport, neutral amino acid transport
4.39	solute carrier family 40 (iron-regulated transporter), member 1	SLC40A1	iron ion transport, cellular iron ion homeostasis, anatomical structure morphogenesis
2.11	solute carrier family 6 (neurotransmitter transporter, taurine), member 6	SLC6A6	amino acid metabolic process, neurotransmitter transport, taurine transport
2.25	SRY (sex determining region Y)-box 4	SOX4	regulation of transcription, DNA-dependent
2.19	serine/threonine kinase 38 like	STK38L	protein amino acid phosphorylation, protein kinase cascade, regulation of cellular component organization and biogenesis
2.13	sulfatase 1	SULF1	apoptosis, heparan sulfate proteoglycan metabolic process
2.17	sulfatase 2	SULF2	heparan sulfate proteoglycan metabolic process
3.36	sushi, von Willebrand factor type A, EGF and pentraxin domain containing 1	SVEP1	cell adhesion
2.58	synaptopodin 2	SYNPO2	
2.87	synaptotagmin XV	SYT15	
3.52	t-complex 11 (mouse)-like 2	TCP11L2	
2.65	transforming growth factor, beta 2	TGFB2	cell morphogenesis, angiogenesis, epithelial to mesenchymal transition, cell-cell signaling, cell death, positive regulation of cell proliferation, extracellular matrix organization and biogenesis, positive regulation of cell growth, neutrophil chemotaxis, wound healing, somatic stem cell division, negative regulation of immune response, positive regulation of immune response
3.81	thrombospondin 2	THBS2	cell adhesion
2.44	thrombospondin 3	THBS3	cell motility, cell-matrix adhesion
2.17	transmembrane 6 superfamily member 1	TM6SF1	no biological data available
2.09	transmembrane protein 155	TMEM155	
2.12	tensin 3	TNS3	
2.72	tumor protein p53 inducible nuclear protein 1	TP53INP1	apoptosis
5.28	tumor protein D52-like 1	TPD52L1	G2/M transition of mitotic cell cycle, DNA fragmentation during apoptosis, induction of apoptosis, positive regulation of MAP kinase activity, positive regulation of JNK cascade
2.26	tetraspanin 2	TSPAN2	cell motility, cell adhesion, cell proliferation
2.11	thioredoxin interacting protein	TXNIP	regulation of transcription, DNA-dependent, keratinocyte differentiation
2.27	very low density lipoprotein	VLDLR	lipid metabolic process, lipid transport, endocytosis,
