# Supplementary material for: Dasatinib reverses Cancer-associated Fibroblasts (CAFs) from primary Lung Carcinomas to a Phenotype comparable to that of normal Fibroblasts
Source: Mol Cancer. 2010 Jun 27;9:168. doi: 10.1186/1476-4598-9-168 (PMC2907332; doi:10.1186/1476-4598-9-168)
Supplement: Additional file 4 — Table S3. Overlap of genes regulated by Dasatinib and upon serum withdrawal [23] [file 1476-4598-9-168-S4.PDF]

Table S3. Overlap of genes regulated by Dasatinib and upon induction of quiescence [23]

Fold change (Dasatinib vs control)	genedescription	gene symbol	Entrez ID	GO biological process
**genes downregulated by Dasatinib**				
3.9609423	cyclin-dependent kinase inhibitor 3 (CDK2-associated dual specificity phosphatase)	CDKN3	1033	regulation of cyclin-dependent protein kinase activity, G1/S transition of mitotic cell cycle, cell cycle arrest, negative regulation of cell proliferation, dephosphorylation
3.7120845		KIAA0101	9768	
3.6725583	discs, large homolog 7 (Drosophila)	DLG7	9787	mitotic chromosome movement towards spindle pole, cell-cell signaling, cell proliferation
3.293451	cell division cycle 20 homolog (S. cerevisiae)	CDC20	991	ubiquitin cycle, cell cycle, mitosis, cell division
3.0957742	CDC28 protein kinase regulatory subunit 2	CKS2	1164	regulation of cyclin-dependent protein kinase activity, cell cycle, spindle organization and biogenesis, meiosis I, cell proliferation, phosphoinositide-mediated signaling, cell division
2.9476745	cyclin B1	CCNB1	891	G2/M transition of mitotic cell cycle, mitosis, cell division
2.939628	lamin B1	LMNB1	4001	
2.905269	thymidylate synthetase	TYMS	7298	dTMP biosynthetic process, DNA replication, DNA repair, phosphoinositide-mediated signaling
2.8938813	forkhead box M1	FOXM1	2305	regulation of transcription, DNA-dependent
2.657287	centromere protein A	CENPA	1058	nucleosome assembly
2.4467447	thymopoietin	TMPO	7112	regulation of transcription
2.4249165	hyaluronan synthase 2	HAS2	3037	
2.4086895	thymidine kinase 1, soluble	TK1	7083	DNA replication
2.3431706	ubiquitin-conjugating enzyme E2S	UBE2S	27338	ubiquitin cycle, regulation of protein metabolic process
2.2158108	stathmin 1/oncoprotein 18	STMN1	3925	microtubule depolymerization, intracellular signaling cascade, multicellular organismal development, nervous system development, cell differentiation
2.1247869	ubiquitin-conjugating enzyme E2S	UBE2S	27338	ubiquitin cycle, regulation of protein metabolic process

Fold change (Dasatinib vs control)	genedescription	gene symbol	Entrez ID	GO biological process
**genes upregulated by Dasatinib**				
4.2540073	complement component 1, s subcomponent	C1S	716	proteolysis, complement activation, classical pathway, G-protein coupled receptor protein signaling pathway
3.971224	complement component 1, r subcomponent	C1R	715	proteolysis, complement activation, classical pathway
3.6401396	actin, alpha 2, smooth muscle, aorta	ACTA2	59	
3.6230714	matrix Gla protein	MGP	4256	cartilage condensation, ossification, multicellular organismal development, response to nutrient, response to mechanical stimulus, response to hormone stimulus, cell differentiation, lung development, regulation of bone mineralization, response to calcium ion
2.7955756	interleukin 6 (interferon, beta 2)	IL6	3569	neutrophil apoptosis, cell-cell signaling, positive regulation of cell proliferation, negative regulation of cell proliferation, negative regulation of apoptosis, positive regulation of MAPKKK cascade, negative regulation of chemokine biosynthetic process
2.6389549	myosin, heavy chain 10, non-muscle	MYH10	4628	cytokinesis after mitosis, regulation of cell shape, actin filament-based movement
2.6090276	alpha-2-macroglobulin	A2M	2	intracellular protein transport, response to nutrient, response to carbon dioxide, protein homooligomerization, response to glucocorticoid stimulus
2.582586	lumican	LUM	4060	visual perception, collagen fibril organization
2.367591	myosin light chain kinase	MYLK	4638	protein amino acid phosphorylation
2.3389912	serpin peptidase inhibitor, clade G (C1 inhibitor), member 1, (angioedema, hereditary)	SERPING 1	710	complement activation, classical pathway, blood coagulation

2.334294	collagen, type III, alpha 1 (Ehlers-Danlos syndrome type IV, autosomal dominant)	COL3A1	1281	phosphate transport, cell-matrix adhesion, transforming growth factor beta receptor signaling pathway, integrin-mediated signaling pathway, peptide cross-linking, platelet activation, collagen fibril organization, collagen biosynthetic process, response to cytokine stimulus, wound healing
2.3108122	epoxide hydrolase 1, microsomal (xenobiotic)	EPHX1	2052	xenobiotic metabolic process, response to toxin, aromatic compound catabolic process
2.2854762	carboxypeptidase E	CPE	1363	proteolysis, neuropeptide signaling pathway, insulin processing
2.210992	glycoprotein (transmembrane) nmb	GPNMB	10457	negative regulation of cell proliferation
2.1874483	nicotinamide N-methyltransferase	NNMT	4837	
2.1842282	inositol 1,4,5-triphosphate receptor, type 1	ITPR1	3708	calcium ion transport, signal transduction
2.1461115	receptor tyrosine kinase-like orphan receptor 1	ROR1	4919	protein amino acid phosphorylation, transmembrane receptor protein tyrosine kinase signaling pathway
2.0948212	enoyl Coenzyme A hydratase 1, peroxisomal	ECH1	1891	generation of precursor metabolites and energy, lipid metabolic process, fatty acid beta-oxidation
2.0496736	low density lipoprotein-related protein 1 (alpha-2-macroglobulin receptor)	LRP1	4035	lipid metabolic process, endocytosis, multicellular organismal development, cell proliferation
