# Supplementary material for: Dasatinib reverses Cancer-associated Fibroblasts (CAFs) from primary Lung Carcinomas to a Phenotype comparable to that of normal Fibroblasts
Source: Mol Cancer. 2010 Jun 27;9:168. doi: 10.1186/1476-4598-9-168 (PMC2907332; doi:10.1186/1476-4598-9-168)
Supplement: Additional file 5 — Table S4. Overlap of genes regulated by Dasatinib with core serum response genes [24] [file 1476-4598-9-168-S5.PDF]

Table S4. Overlap of genes regulated by Dasatinib with core serum response genes [24]

Fold change (Dasatinib vs control)	gene description	gene symbol	Entrez ID	GO biological process
**genes downregulated by Dasatinib**				
3.2596993	integrin, alpha 6	ITGA6	3655	cell-substrate junction assembly, cell adhesion, cell-matrix adhesion, integrin-mediated signaling pathway
2.9425588	chromosome 6 open reading frame 173	C6orf173	387103	
2.776894	replication factor C (activator 1) 3, 38kDa	RFC3	5983	DNA strand elongation during DNA replication
2.7354112	chromosome 18 open reading frame 24	C18orf24	220134	cell cycle, mitosis, cell division
2.7197442	MLF1 interacting protein	MLF1IP	79682	regulation of transcription, DNA-dependent
2.6691477	transmembrane protein 48	TMEM48	55706	nuclear pore distribution, mRNA transport, nuclear pore complex assembly, intracellular protein transport across a membrane
2.6458812	structural maintenance of chromosomes 2	SMC2	10592	DNA metabolic process, cell cycle, mitotic chromosome condensation, cell division
2.632881	polymerase (DNA directed), epsilon 2 (p59 subunit)	POLE2	5427	DNA replication, DNA repair
2.52232	arylacetamide deacetylase-like 1	AADACL1	57552	metabolic process
2.5178578	centromere protein O	CENPO	79172	
2.5000436	centromere protein N	CENPN	55839	
2.4974368	minichromosome maintenance complex component 7	MCM7	4176	DNA replication initiation, regulation of transcription, DNA-dependent, response to DNA damage stimulus, cell cycle, regulation of phosphorylation
2.4667864	H2A histone family, member Z	H2AFZ	3015	nucleosome assembly
2.4597127	breast cancer 2, early onset	BRCA2	675	DNA damage response, signal transduction by p53 class mediator resulting in transcription of p21 class mediator, cell aging, response to X-ray, response to UV-C, response to gamma radiation, mammary gland development, cytokinesis during cell cycle, response to estrogen stimulus, positive regulation of mitotic cell cycle, replication fork protection
2.4465199	dihydrofolate reductase	DHFR	1719	glycine biosynthetic process, nucleotide biosynthetic process
2.4398885	WD repeat and HMG-box DNA binding protein 1	WDHD1	11169	regulation of transcription, DNA-dependent
2.432511	minichromosome maintenance complex component 3	MCM3	4172	DNA replication initiation, regulation of transcription, DNA-dependent, cell cycle
2.4249165	hyaluronan synthase 2	HAS2	3037	

2.2610013	cyclin-dependent kinase 2	CDK2	1017	G2/M transition of mitotic cell cycle, regulation of DNA replication, protein amino acid phosphorylation, cell cycle, mitosis, traversing start control point of mitotic cell cycle, positive regulation of cell proliferation, cell division
2.2314289	ribonuclease H2, subunit A	RNASEH2A	10535	DNA replication, RNA catabolic process
2.1541805	replication factor C (activator 1) 5, 36.5kDa	RFC5	5985	DNA replication, DNA repair
2.1474488	v-myb myeloblastosis viral oncogene homolog (avian)-like 2	MYBL2	4605	regulation of transcription, DNA-dependent, transcription from RNA polymerase II promoter, anti-apoptosis, multicellular organismal development
2.1359878	tyrosyl-DNA phosphodiesterase 1	TDP1	55775	DNA repair
2.0926628	chromosome 13 open reading frame 27	C13orf27	93081	
2.0164568	v-myb myeloblastosis viral oncogene homolog (avian)-like 1	MYBL1	4603	regulation of transcription, DNA-dependent
2.0098922	discoidin, CUB and LCCL domain containing 2	DCBLD2	131566	cell adhesion, negative regulation of cell growth, intracellular receptor-mediated signaling pathway, wound healing

Fold change (Dasatinib vs control)	gene description	gene symbol	Entrez ID	GO biological process
**genes upregulated by Dasatinib**				
4.3914075	solute carrier family 40 (iron-regulated transporter), member 1	SLC40A1	30061	iron ion transport, cellular iron ion homeostasis, anatomical structure morphogenesis
4.2970023	chromosome 1 open reading frame 198	C1orf198	84886	
3.896681	GABA(A) receptor-associated protein like 1	GABARAPL 1	23710	
3.3624842	sushi, von Willebrand factor type A, EGF and pentraxin domain containing 1	SVEP1	79987	cell adhesion
3.270258	nuclear protein 1	NUPR1	26471	induction of apoptosis, cell growth
2.904519	kelch-like 24 (Drosophila)	KLHL24	54800	
2.7236748	tumor protein p53 inducible nuclear protein 1	TP53INP1	94241	apoptosis
2.582586	lumican	LUM	4060	visual perception, collagen fibril organization
2.5752075	protein-L-isoaspartate (D-aspartate) O-methyltransferase domain containing 1	PCMTD1	115294	protein modification process
2.4478831	chromosome 10 open reading frame 10	C10orf10	11067	
2.3389912	serpin peptidase inhibitor, clade G (C1 inhibitor), member 1, (angioedema, hereditary)	SERPING1	710	complement activation, classical pathway, blood coagulation, blood circulation
2.1546316	milk fat globule-EGF factor 8 protein	MFGE8	4240	cell adhesion, single fertilization
2.1401227	jagged 1 (Alagille syndrome)	JAG1	182	angiogenesis, morphogenesis of an epithelial sheet, cell communication, multicellular organismal development, nervous system development, keratinocyte differentiation, regulation of cell migration, regulation of cell proliferation, myoblast differentiation, endothelial cell differentiation, negative regulation of cell differentiation, positive regulation of myeloid cell differentiation, positive regulation of Notch signaling pathway
2.0741422	chromosome 4 open reading frame 18	C4orf18	51313	
2.0542133	plasma glutamate carboxypeptidase	PGCP	10404	proteolysis
2.0496736	low density lipoprotein-related protein 1 (alpha-2-macroglobulin receptor)	LRP1	4035	lipid metabolic process, endocytosis, multicellular organismal development, cell proliferation
2.0041502	calcium binding and coiled-coil domain 1	CALCOCO1	57658	transcription, signal transduction, Wnt receptor signaling pathway, steroid hormone receptor signaling pathway
