# Supplementary material for: Dasatinib reverses Cancer-associated Fibroblasts (CAFs) from primary Lung Carcinomas to a Phenotype comparable to that of normal Fibroblasts
Source: Mol Cancer. 2010 Jun 27;9:168. doi: 10.1186/1476-4598-9-168 (PMC2907332; doi:10.1186/1476-4598-9-168)
Supplement: Additional file 6 — Table S5. Overlap of genes regulated by Dasatinib with genes differentially expressed in NAFs vs. CAFs [26] [file 1476-4598-9-168-S6.PDF]

Table S5. Overlap of genes regulated by Dasatinib with genes differentially expressed in NAFs vs. CAFs [26]

Fold change (Dasatinib vs control)	gene description	gene symbol	Entrez ID	GO biological process
**genes downregulated by Dasatinib**				
3.9609423	cyclin-dependent kinase inhibitor 3 (CDK2-associated dual specificity phosphatase)	CDKN3	1033	regulation of cyclin-dependent protein kinase activity, G1/S transition of mitotic cell cycle, cell cycle arrest, negative regulation of cell proliferation, dephosphorylation
3.7120845		KIAA0101	9768	
3.6917882		TTK	7272	protein amino acid phosphorylation, mitotic spindle organization and biogenesis, mitotic cell cycle spindle assembly checkpoint, positive regulation of cell proliferation
3.6725583	discs, large homolog 7 (Drosophila)	DLG7	9787	mitotic chromosome movement towards spindle pole, cell-cell signaling, cell proliferation
3.6318128	serpin peptidase inhibitor, clade B (ovalbumin), member 2	SERPINB2	5055	anti-apoptosis
3.6045253	endothelial cell-specific molecule 1	ESM1	11082	regulation of cell growth
3.5937507	matrix metallopeptidase 1 (interstitial collagenase)	MMP1	4312	proteolysis, metabolic process, collagen catabolic process
3.5379066	apolipoprotein B mRNA editing enzyme, catalytic polypeptide-like 3B	APOBEC3B	9582	no biological data available
3.4784048	antigen identified by monoclonal antibody Ki-67	MKI67	4288	cell cycle, cell proliferation
3.4780145	cell division cycle 6 homolog (S. cerevisiae)	CDC6	990	DNA replication checkpoint, regulation of cyclin-dependent protein kinase activity, traversing start control point of mitotic cell cycle, negative regulation of DNA replication, negative regulation of cell proliferation, cell division
3.4623456	polo-like kinase 4 (Drosophila)	PLK4	10733	protein amino acid phosphorylation
3.293451	cell division cycle 20 homolog (S. cerevisiae)	CDC20	991	ubiquitin cycle, cell cycle, mitosis, cell division
3.2611222		NDC80	10403	mitotic sister chromatid segregation, cell cycle, spindle organization and biogenesis, phosphoinositide-mediated signaling, cell division
3.2422917	cyclin B2	CCNB2	9133	cell cycle, mitosis, cell division
3.227939	ribonucleotide reductase M2 polypeptide	RRM2	6241	DNA replication
3.2190335	cell division cycle 2, G1 to S and G2 to M	CDC2	983	protein amino acid phosphorylation, anti-apoptosis, cell cycle, traversing start control point of mitotic cell cycle, cell division
3.166044		TPX2	22974	mitosis, cell proliferation
3.1487994	kinesin family member 11	KIF11	3832	microtubule-based movement, cell cycle, mitotic centrosome separation, spindle pole body organization and biogenesis, cell division

3.142741	kinesin family member 2C	KIF2C	11004	microtubule-based movement, mitosis, cell proliferation, establishment and/or maintenance of microtubule cytoskeleton polarity
3.131805	ZW10 interactor	ZWINT	11130	mitotic sister chromatid segregation, spindle organization and biogenesis, mitotic cell cycle checkpoint, phosphoinositide-mediated signaling, cell division, establishment of localization in cell
3.1270027		SPC25	57405	cell cycle, mitosis, cell division
3.0957742	CDC28 protein kinase regulatory subunit 2	CKS2	1164	regulation of cyclin-dependent protein kinase activity, cell cycle, spindle organization and biogenesis, meiosis I, cell proliferation, phosphoinositide-mediated signaling, cell division
3.016271	aurora kinase A	AURKA	6790	protein amino acid phosphorylation, cell cycle, spindle organization and biogenesis, mitosis, regulation of protein stability, phosphoinositide-mediated signaling
2.9476745	cyclin B1	CCNB1	891	G2/M transition of mitotic cell cycle, cell division
2.926012	kinesin family member C1	KIFC1	3833	mitotic sister chromatid segregation, microtubule-based movement, cell cycle, cell division
2.9176307	thyroid hormone receptor interactor 13	TRIP13	9319	transcription from RNA polymerase II promoter
2.905269	thymidylate synthetase	TYMS	7298	dTMP biosynthetic process, DNA replication, DNA repair, phosphoinositide-mediated signaling
2.8991559	topoisomerase (DNA) II alpha 170kDa	TOP2A	7153	DNA replication, DNA ligation, DNA repair, chromosome segregation, apoptotic chromosome condensation, positive regulation of retroviral genome replication
2.8938813	forkhead box M1	FOXM1	2305	regulation of transcription, DNA-dependent
2.8046918	GINS complex subunit 1 (Psf1 homolog)	GINS1	9837	inner cell mass cell proliferation, DNA replication
2.7687316	minichromosome maintenance complex component 5	MCM5	4174	DNA replication initiation, regulation of transcription, DNA-dependent
2.6595068	ectonucleotide pyrophosphatase/phosphodie sterase 1	ENPP1	5167	ossification, generation of precursor metabolites and energy, phosphate metabolic process, nucleoside triphosphate catabolic process, negative regulation of cell growth, regulation of bone mineralization, inorganic diphosphate transport, sequestering of triacylglycerol, negative regulation of protein amino acid autophosphorylation, cellular response to insulin stimulus, negative regulation of fat cell differentiation, negative regulation of glycogen biosynthetic process, negative regulation of glucose import, negative regulation of insulin receptor signaling pathway
2.657287	centromere protein A	CENPA	1058	nucleosome assembly
2.6500502	high mobility group AT-hook 2	HMGA2	8091	regulation of cell growth, establishment and/or maintenance of chromatin architecture, regulation of transcription, DNA-dependent, mitosis, multicellular organismal development, cell division

2.6458812	structural maintenance of chromosomes 2	SMC2	10592	DNA metabolic process, cell cycle, mitotic chromosome condensation, chromosome organization and biogenesis, cell division
2.632881	polymerase (DNA directed), epsilon 2 (p59 subunit)	POLE2	5427	DNA replication, DNA repair
2.6157975	G-protein signaling modulator 2 (AGS3-like, C. elegans)	GPSM2	29899	signal transduction, G-protein coupled receptor protein signaling pathway
2.5585783	flap structure-specific endonuclease 1	FEN1	2237	DNA replication, double-strand break repair, UV protection, phosphoinositide-mediated signaling
2.5429876	ubiquitin-conjugating enzyme E2C	UBE2C	11065	cell cycle, spindle organization and biogenesis, mitosis, cyclin catabolic process, protein ubiquitination, positive regulation of exit from mitosis, postranslational protein modification, phosphoinositide-mediated signaling, cell division
2.5426474	Rho GTPase activating protein 22	ARHGAP22	58504	angiogenesis, regulation of transcription, DNA-dependent, signal transduction, multicellular organismal development, cell differentiation
2.5216281	PSMC3 interacting protein	PSMC3IP	29893	DNA recombination, meiosis
2.5123243	polymerase (DNA directed), alpha 2 (70kD subunit)	POLA2	23649	protein import into nucleus, translocation, DNA replication
2.5070863	dihydrofolate reductase	DHFR	1719	glycine biosynthetic process, nucleotide biosynthetic process
2.4974368	minichromosome maintenance complex component 7	MCM7	4176	DNA replication initiation, regulation of transcription, DNA-dependent, response to DNA damage stimulus, cell cycle, regulation of phosphorylation
2.4540908	leupaxin	LPXN	9404	protein complex assembly, cell adhesion, signal transduction
2.4431622	histone cluster 1, H1a	HIST1H1A	3024	nucleosome assembly, spermatogenesis
2.4282608	enhancer of zeste homolog 2 (Drosophila)	EZH2	2146	establishment and/or maintenance of chromatin architecture, regulation of transcription, DNA-dependent
2.4123294	neurofilament, medium polypeptide 150kDa	NEFM	4741	axon cargo transport, regulation of axon diameter, intermediate filament bundle assembly, neurofilament cytoskeleton organization and biogenesis
2.4086895	thymidine kinase 1, soluble	TK1	7083	DNA replication
2.3691812	aurora kinase B	AURKB	9212	cytokinesis, protein amino acid phosphorylation, cell cycle, mitosis
2.362405	baculoviral IAP repeat-containing 5 (survivin)	BIRC5	332	G2/M transition of mitotic cell cycle, cytokinesis, apoptosis, anti-apoptosis, protein complex localization, positive regulation of exit from mitosis, spindle checkpoint, negative regulation of caspase activity, positive regulation of mitotic cell cycle, establishment of chromosome localization
2.3543415	kinesin family member 23	KIF23	9493	mitotic spindle elongation, microtubule-based movement, cell cycle, mitosis, cell division
2.344488	G-2 and S-phase expressed 1	GTSE1	51512	G2 phase of mitotic cell cycle, DNA damage response, signal transduction by p53 class mediator resulting in cell cycle arrest, microtubule-based process

2.344163	replication factor C (activator 1) 4, 37kDa	RFC4	5984	DNA strand elongation during DNA replication, DNA repair, phosphoinositide-mediated signaling
2.3143635	high-mobility group box 2	HMGB2	3148	DNA unwinding during replication, DNA repair, base-excision repair, DNA ligation, establishment and/or maintenance of chromatin architecture, regulation of transcription from RNA polymerase II promoter, phosphoinositide-mediated signaling
2.2724392	minichromosome maintenance complex component 6	MCM6	4175	DNA unwinding during replication, DNA replication initiation, regulation of transcription, DNA-dependent, cell cycle
2.2314289	ribonuclease H2, subunit A	RNASEH2A	10535	DNA replication, RNA catabolic process
2.2158108	stathmin 1/oncoprotein 18	STMN1	3925	microtubule depolymerization, mitotic spindle organization and biogenesis, intracellular signaling cascade, multicellular organismal development, nervous system development, cell differentiation
2.2028444	dickkopf homolog 1 (Xenopus laevis)	DKK1	22943	multicellular organismal development, negative regulation of Wnt receptor signaling pathway, embryonic limb morphogenesis
2.1541805	replication factor C (activator 1) 5, 36.5kDa	RFC5	5985	DNA replication, DNA repair
2.1474488	v-myb myeloblastosis viral oncogene homolog (avian)-like 2	MYBL2	4605	regulation of transcription, DNA-dependent, transcription from RNA polymerase II promoter, anti-apoptosis, multicellular organismal development
2.1311193		RAD51	5888	double-strand break repair via homologous recombination, DNA unwinding during replication, mitotic recombination, meiotic recombination, positive regulation of DNA ligation, protein homooligomerization
2.0704875	DEAD (Asp-Glu-Ala-Asp) box polypeptide 39	DDX39	10212	nuclear mRNA splicing, via spliceosome, mRNA export from nucleus
2.0466595	plasminogen activator, urokinase	PLAU	5328	proteolysis, chemotaxis, signal transduction, blood coagulation, fibrinolysis

Fold change (Dasatinib vs control)	gene description	gene symbol	Entrez ID	GO biological process
**genes upregulated by Dasatinib**				
6.3464217	microfibrillar-associated protein 4	MFAP4	4239	cell adhesion, signal transduction
4.2540073	complement component 1, s subcomponent	C1S	716	proteolysis, complement activation, classical pathway, G-protein coupled receptor protein signaling pathway
3.971224	complement component 1, r subcomponent	C1R	715	proteolysis, complement activation, classical pathway
3.3624842	sushi, von Willebrand factor type A, EGF and pentraxin domain containing 1	SVEP1	79987	cell adhesion
3.270258	nuclear protein 1	NUPR1	26471	induction of apoptosis, cell growth
3.1223207	fibulin 5	FBLN5	10516	cell adhesion, cell-matrix adhesion, blood coagulation
2.6389549	myosin, heavy chain 10, non-muscle	MYH10	4628	cytokinesis after mitosis, regulation of cell shape, actin filament-based movement
2.6265175	UDP-Gal:betaGlcNAc beta 1,3-galactosyltransferase, polypeptide 2	B3GALT2	8707	protein amino acid glycosylation, oligosaccharide biosynthetic process
2.5850434	asparagine synthetase	ASNS	440	asparagine biosynthetic process, glutamine metabolic process, cellular response to glucose starvation, negative regulation of apoptosis, positive regulation of mitotic cell cycle
2.5462976	platelet-derived growth factor receptor, beta polypeptide	PDGFRB	5159	protein amino acid phosphorylation, signal transduction, transmembrane receptor protein tyrosine kinase signaling pathway, regulation of peptidyl-tyrosine phosphorylation
2.4478831	chromosome 10 open reading frame 10	C10orf10	11067	
2.4424756	thrombospondin 3	THBS3	7059	cell motility, cell adhesion, cell-matrix adhesion
2.4072123	sarcoglycan, delta (35kDa dystrophin-associated glycoprotein)	SGCD	6444	cytoskeleton organization and biogenesis, muscle development
2.367591	myosin light chain kinase	MYLK	4638	protein amino acid phosphorylation
2.2952852	HEG homolog 1 (zebrafish)	HEG1	57493	
2.2815335	phosphoenolpyruvate carboxykinase 2 (mitochondrial)	PCK2	5106	gluconeogenesis
2.2515926	ATP-binding cassette, sub-family C (CFTR/MRP), member 3	ABCC3	8714	transport
2.1874483	nicotinamide N-methyltransferase	NNMT	4837	
2.1417515	prostaglandin I2 (prostacyclin) synthase	PTGIS	5740	prostaglandin biosynthetic process, fatty acid biosynthetic process
2.139726	HtrA serine peptidase 1	HTRA1	5654	regulation of cell growth, proteolysis, negative regulation of transforming growth factor beta receptor signaling pathway, negative regulation of BMP signaling pathway

2.1296947	sulfatase 1	SULF1	23213	apoptosis, metabolic process, heparan sulfate proteoglycan metabolic process
2.0886462	furry homolog (Drosophila)	FRY	10129	regulation of transcription, DNA-dependent
2.0769942	ataxin 1	ATXN1	6310	RNA processing, cell death, adult locomotory behavior, visual learning, negative regulation of transcription, nuclear export, regulation of excitatory postsynaptic membrane potential
2.020234	argininosuccinate synthetase 1	ASS1	445	urea cycle, arginine biosynthetic process
2.016423	tryptophanyl-tRNA synthetase	WARS	7453	tryptophanyl-tRNA aminoacylation, negative regulation of cell proliferation
2.0041502	calcium binding and coiled-coil domain 1	CALCOCO1	57658	transcription, signal transduction, Wnt receptor signaling pathway, steroid hormone receptor signaling pathway
