# Supplementary figures and images for: Dasatinib reverses Cancer-associated Fibroblasts (CAFs) from primary Lung Carcinomas to a Phenotype comparable to that of normal Fibroblasts
Source: Mol Cancer. 2010 Jun 27;9:168. doi: 10.1186/1476-4598-9-168 (PMC2907332; doi:10.1186/1476-4598-9-168)

Figure S1

Array

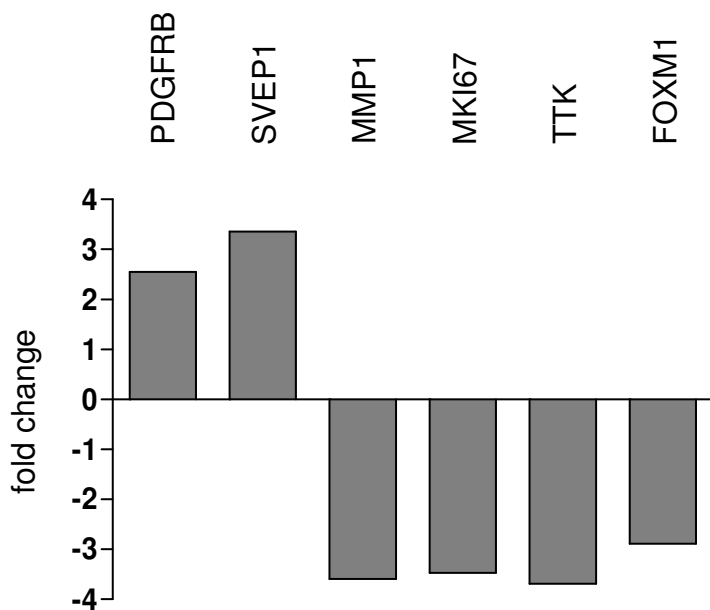

PCR

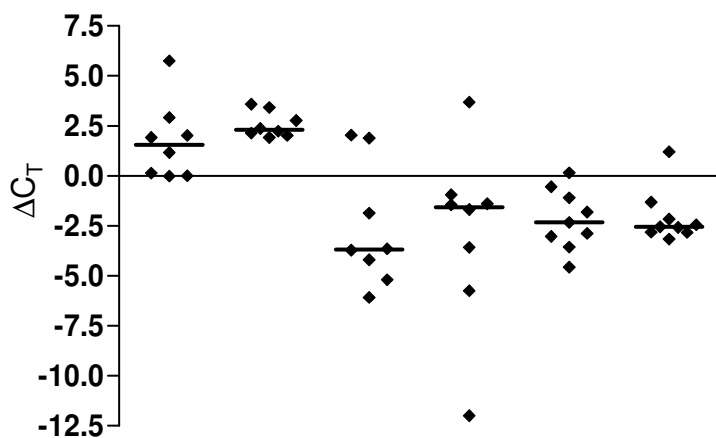

Supplement: Additional file 7 — Figure S1. qRT-PCR validation of 6 genes from the overlap of genes regulated by Dasatinib with differentially regulated genes in NAFs vs. CAFs [26]. Upper panel: graphical presentation of expression array data for six differentially expressed genes selected for qRT-PCR validation. Mean fold change of nine CAF cultures upon Dasatinib treatment. Lower panel: Expression levels (delta CT values) of selected genes as assessed by qRT-PCR in eight CAF cultures. Significant differences in expression in Dasatinib treated CAFs and controls were found for PDGFR (p = 0.039), SVEP1 (p < 0.0001), MMP1 (p = 0.048), TTK (p = 0.003), and FOXM1 (p = 0.002). [file 1476-4598-9-168-S7.PDF]
